# Theoretical and NMR Conformational Studies of β-Proline Oligopeptides With Alternating Chirality of Pyrrolidine Units

**DOI:** 10.3389/fchem.2018.00091

**Published:** 2018-03-28

**Authors:** Alexey B. Mantsyzov, Oleg Y. Savelyev, Polina M. Ivantcova, Stefan Bräse, Konstantin V. Kudryavtsev, Vladimir I. Polshakov

**Affiliations:** ^1^Faculty of Fundamental Medicine, M.V. Lomonosov Moscow State University, Moscow, Russia; ^2^Department of Chemistry, M.V. Lomonosov Moscow State University, Moscow, Russia; ^3^Institute of Organic Chemistry, Karlsruhe Institute of Technology, Karlsruhe, Germany; ^4^Institute of Toxicology and Genetics, Karlsruhe Institute of Technology, Eggenstein-Leopoldshafen, Germany; ^5^Institute of Physiologically Active Compounds, Russian Academy of Sciences, Chernogolovka, Russia

**Keywords:** β-peptides, folding, NMR spectroscopy, solution structure, restrained molecular dynamics, density functional theory (DFT) calculations

## Abstract

Synthetic β-peptides are potential functional mimetics of native α-proteins. A recently developed, novel, synthetic approach provides an effective route to the broad group of β-proline oligomers with alternating patterns of stereogenic centers. Conformation of the pyrrolidine ring, *Z*/*E* isomerism of β-peptide bonds, and hindered rotation of the neighboring monomers determine the spatial structure of this group of β-proline oligopeptides. Preferences in their structural organization and corresponding thermodynamic properties are determined by NMR spectroscopy, restrained molecular dynamics and quantum mechanics. The studied β-proline oligopeptides exist in dimethyl sulfoxide solution in a limited number of conformers, with compatible energy of formation and different spatial organization. In the β-proline tetrapeptide with alternating chirality of composing pyrrolidine units, one of three peptide bonds may exist in an *E* configuration. For the alternating β-proline pentapeptide, the presence of an *E* configuration for at least of one β-peptide bond is mandatory. In this case, three peptide bonds synchronously change their configurations. Larger polypeptides may only exist in the presence of several *E* configurations of β-peptide bonds forming a wave-like extended structure.

## Introduction

Well-conceived and synthesized as structural homologs and potential functional mimetics of α-proteins, β-peptides have become a valuable tool for understanding molecular assembly and practical applications (Cheng et al., 2001; Seebach and Gardiner, 2008). β-Peptides are able to adopt stable secondary structures; specific folding is the most prominent physical property of these artificial oligomers (Gellman, 1998). Considered as substitutes for native α-peptide sequences, β-peptides have been developed as ligands with improved properties for receptors, inhibitors of protein-protein interactions and membrane-targeting agents (Cabrele et al., 2014). Besides biological activity, the metabolic stability, together with the predictable side-chain topography of β-peptides has been exploited in the design of self-assembled proteomimetic bundles and nanomaterials (Gopalan et al., 2015). Helical, sheet and strand secondary structures stabilized by intramolecular hydrogen bonds are documented for short β-peptides (Cheng et al., 2001). A special class of β-peptide oligomers constitutes non-hydrogen bonded β-polyprolines (Huck et al., 1999, 2003; Kim et al., 2000).

Polyproline peptides are prone to two main types of structural elements: an extended PPII left-handed helix conformation with a *trans* configuration of a peptide bond, formed in aqueous solution (Doose et al., 2007), and a more compact PPI right-handed helix with a *cis*-peptide bond formed in aliphatic alcohols such as MeOH or PrOH (Moradi et al., 2009). The PPI helix is able to pass to PPII in a cascade fashion, with a sequential flip of *cis* to *trans* peptide bonds starting from the N-terminus (Shi et al., 2014). PPII helix motifs minimize the entropic costs of binding and conformational strain in different protein-protein complexes (Ball et al., 2005). Formation of PPII helices was recently demonstrated for oligomers constituted of β-proline units (Caumes et al., 2013). It was shown that some 4-substituted proline polypeptides, depending on solution and/or pH, may exist in either PPII form or in a non-classical antiparallel β-structure (Sonar and Ganesh, 2010) capable to form nanofibers (Bansode et al., 2016). Several attempts to achieve certain conformational control by substitution or bridging of pyrrolidine rings in the β-polyproline scaffold were reported (Otani et al., 2006; Krow et al., 2010; Wang et al., 2014). Circular dichroism (CD) (Huck et al., 1999; Kim et al., 2000; Caumes et al., 2013), X-ray crystallography (Krow et al., 2010; Kudryavtsev et al., 2013, 2015a; Wang et al., 2014), NMR spectroscopy (Huck et al., 2003; Krow et al., 2010; Caumes et al., 2013; Wang et al., 2014; Kudryavtsev et al., 2015a,b) and quantum mechanics calculation (Otani et al., 2006, 2017; Wang et al., 2014) revealed the formation of ordered structures in solution and solid state for the reported β-proline oligopeptides and indicated the presence of an equilibrium between several dominant conformations in solution.

**Figure 1:**
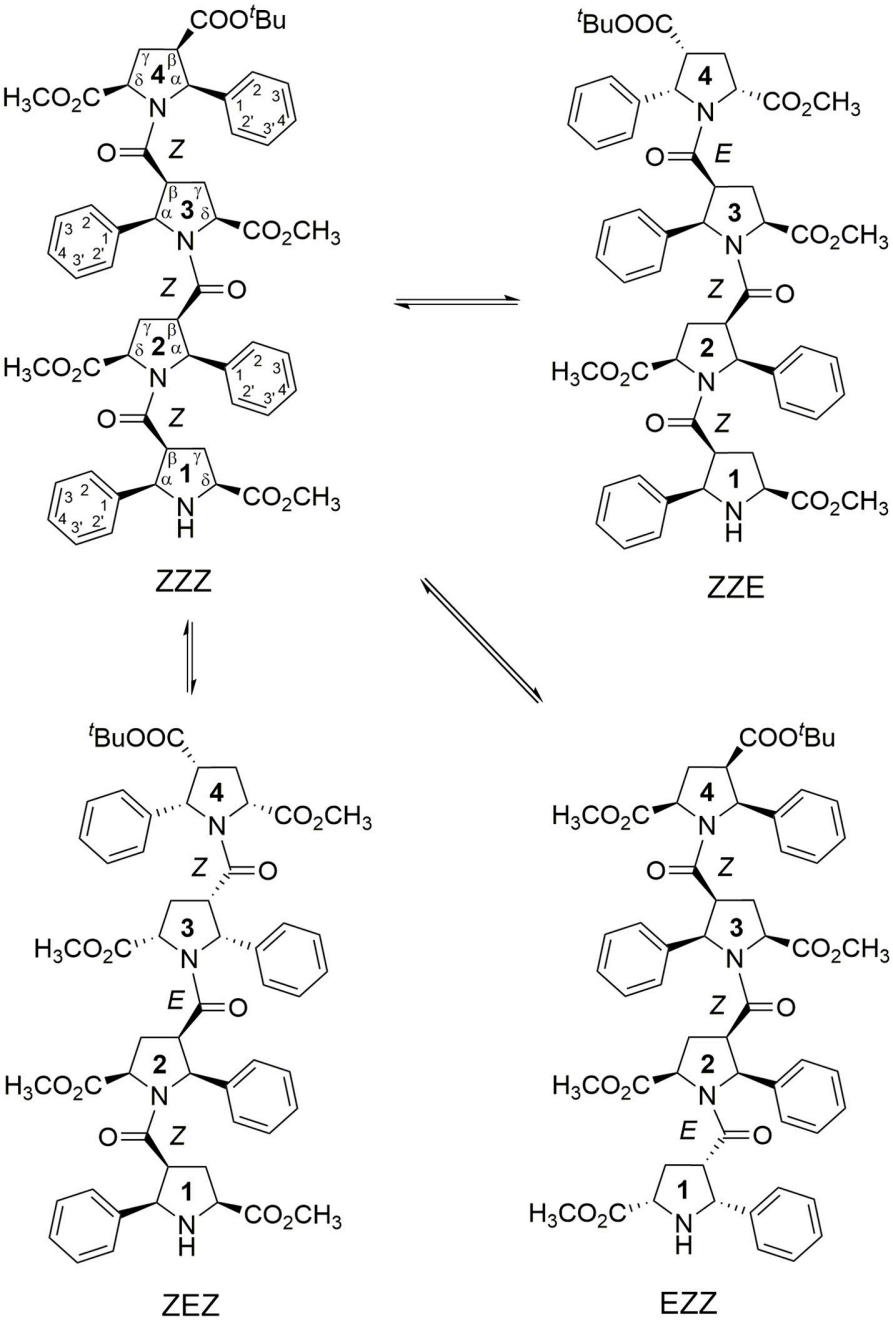
Four main conformations detected in solution for the racemic alternating β-proline tetrapeptide **1**. Shown are residues numbering, atom notations and relative configurations of stereogenic centers.

**Figure 2:**
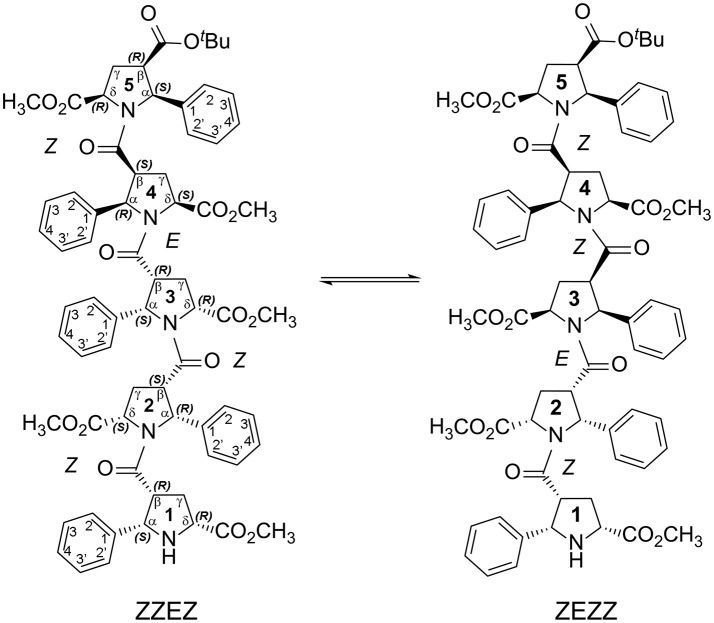
Two main conformations detected in solution for the enantiomerically pure alternating β-proline pentapeptide **2**. Shown are residues numbering, atom notations and absolute configurations of stereogenic centers.

We developed an exceptionally novel synthetic approach to β-proline oligopeptides with the alternating patterns of a β-peptide scaffold (Kudryavtsev et al., 2013, 2015a). Insertion of a bulky phenyl ring at the Cα position and an ester group at Cδ position of pyrrolidine rings in the β-proline polypeptide scaffold (Figures 1, 2) results in formation of several stable conformers primarily due to β-peptide bond isomerization (Kudryavtsev et al., 2013, 2015a,b). Promising anti-cancer properties were also demonstrated for this specific class of β-peptides (Kudryavtsev et al., 2015a,b; Chan et al., 2017).

We present here the results of our NMR and quantum mechanical studies of well-defined alternating β-proline oligomers in solution since the previous efforts (Kudryavtsev et al., 2013, 2015a,b) to determine secondary structure preferences for the novel β-peptide class in solution did not provide necessary details. NMR-derived distance constraints allowed us to determine structure in solution for all the major conformations observed for tetrameric and pentameric species of alternating β-proline polypeptides (Figures 1, 2). We determined distinct conformational preferences to form (*ZZ*)-segments in an alternating β-polyproline pattern that in the case of tetramer **1** led to folding in a right-handed coil with well-organized periphery substituents. Also, other alternating β-polyproline chain degrees of freedom were studied in detail, such as the configuration of the peptide bond (*cis* or *trans*), conformation of the pyrrolidine ring, and rotation around the Cβ-C(O) bond.

## Materials and methods

All studied compounds were synthesized as previously described (Kudryavtsev et al., 2013). Racemic β-tetrapeptide **1** and enantiomerically pure β-pentapeptide **2** were used for subsequent studies. NMR spectra were recorded on a Bruker AVANCE spectrometer operating at a proton frequency of 600 MHz, at 298K in DMSD-d_6_ at a concentration of samples of 5–7 mM, with TMS as an internal reference. Mixing times for TOCSY, ROESY and NOESY experiments were 70, 300, and 350 ms respectively. Spectra were processed by NMRPipe (Delaglio et al., 1995) using standard protocol that includes the Lorentz-to-Gauss window function, forward-backward linear prediction and polynomial baseline correction, and analyzed with NMRFAM-Sparky (Lee et al., 2015). Experimental details of NMR measurements and ^1^H and ^13^C signal assignments were reported earlier (Kudryavtsev et al., 2015b). Most of the NOE distance restraints were obtained from the ROESY spectra (Supplementary Figure 1). ROE peaks were integrated and calibrated, and corresponding distances have been clustered in four groups with upper inter-proton distance of 2.5, 3.5, 4.5, and 5.5 Å. Volumes of Hα-Hβ cross peaks were used for ROE calibration, as the distance between Hα and Hβ atoms constitutes approximately ~2.3Å in both exo and endo conformations of the pyrrolidine ring.

Structure calculation and refinement was performed using CNS software (Brunger et al., 1998). Topology and parameter files were prepared using a PRODRG server (Schuttelkopf and van Aalten, 2004) and manually checked and corrected. The refinement protocol included a high-temperature Cartesian molecular dynamic phase (15 ps at *T* = 50,000 K) followed by a slow-cooling Cartesian phase (temperature decreased from 1,000 to 0 K), and finally a Powell energy minimization (20 cycles of 400 steps). The final structure families for each conformer contained 20 models with no distance restraint violation larger than 0.3 Å. For each NMR family (Supplementary Figure 2), a representative structure was selected using the criterion of having the lowest sum of the pairwise RMSD for the remaining structures in the family. Coordinates of atoms, chemical shift assignments and NMR restraints were deposited to BioMagResBank (http://www.bmrb.wisc.edu) under the accession numbers BMRB-21065 (*ZZZ*- tetrapeptide **1**), 21067 (*ZZE*-tetrapeptide **1**), 21066 (*ZEZ*-tetrapeptide **1**), 21064 (*EZZ*-tetrapeptide **1**), 21068 (*ZZEZ*-pentapeptide **2**), and 21069 (*ZEZZ*-pentapeptide **2**).

Geometries of each representative structure of tetrapeptide **1** were additionally optimized by Density Functional Theory (DFT) quantum mechanical calculations using the Gaussian 09w suite (Frisch et al., 2009) (Supplementary Figure 3). Calculations were performed by DFT using the B3LYP functional and 6-31+G(d,p) basis set and the Polarizable Continuum Model (PCM) of dimethyl sulfoxide solvent. Representative structures of two pentapeptide **2** conformers (Supplementary Figure 4) were optimized at the Hartree-Fock level using the 6-31G(d,p) basis set. Relaxed potential energy scans between *Z* and *E* isomers and for rotation around C(O)-Cβ bond were performed by DFT using B3LYP functional and 6-311+G(d,p) basis set with 6 or 10° step of corresponding dihedral angle change and full geometry optimization of each intermediate structure.

^1^H and ^13^C chemical shifts of tetrapeptide **1** conformers were calculated for each optimized model using the GIAO (Gauge Independent Atomic Orbitals) method, the mPW1PW91 functional (Adamo and Barone, 1998), the 6-311+G(2d,p) basis set and the Polarizable Continuum Model (PCM) of dimethyl sulfoxide solvent. ^1^H and ^13^C chemical shifts were computed from the isotropic values of SCF GIAO magnetic shielding tensors using linear regression (Lodewyk et al., 2012) (Tables S1–S4). ^1^H-^1^H coupling constants were calculated by DFT using B3LYP functional and a 6-31+G(d,p) basis set.

Structure visualization and analysis were carried out using the PyMol (Schrodinger, 2015), InsightII and Discovery Studio (Dassault Systèmes BIOVIA, San Diego, USA) and Molden (Schaftenaar and Noordik, 2000) software.

## Results and discussion

### Conformations of β-proline oligopeptides

NMR signal assignments for four conformers of the alternating β-proline tetrapeptide **1** and two conformers of the alternating β-proline pentapeptide **2** have been previously published (Kudryavtsev et al., 2015b). Both β-peptides exist in several conformational states interconverting slowly in the NMR time scale (Figures 1, 2).

#### *Z* and *E* peptide bond configuration

**Figure 3:**
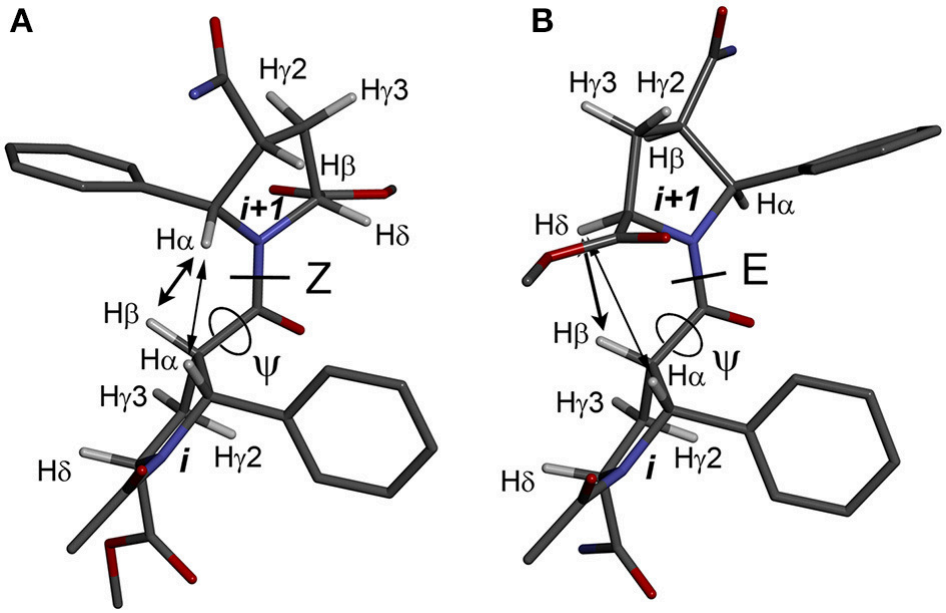
Structures of the alternating β-proline dimeric fragment with *Z* **(A)** and *E* **(B)** configuration of the β-peptide bond, calculated using DFT. NOEs between Hβ_i_ and Hα_i+1_ in *Z* isomer, and Hβ_i_ and Hδ_i+1_ in *E* isomer, are shown by arrows. Dihedral angles ψ are labeled.

**Figure 4:**
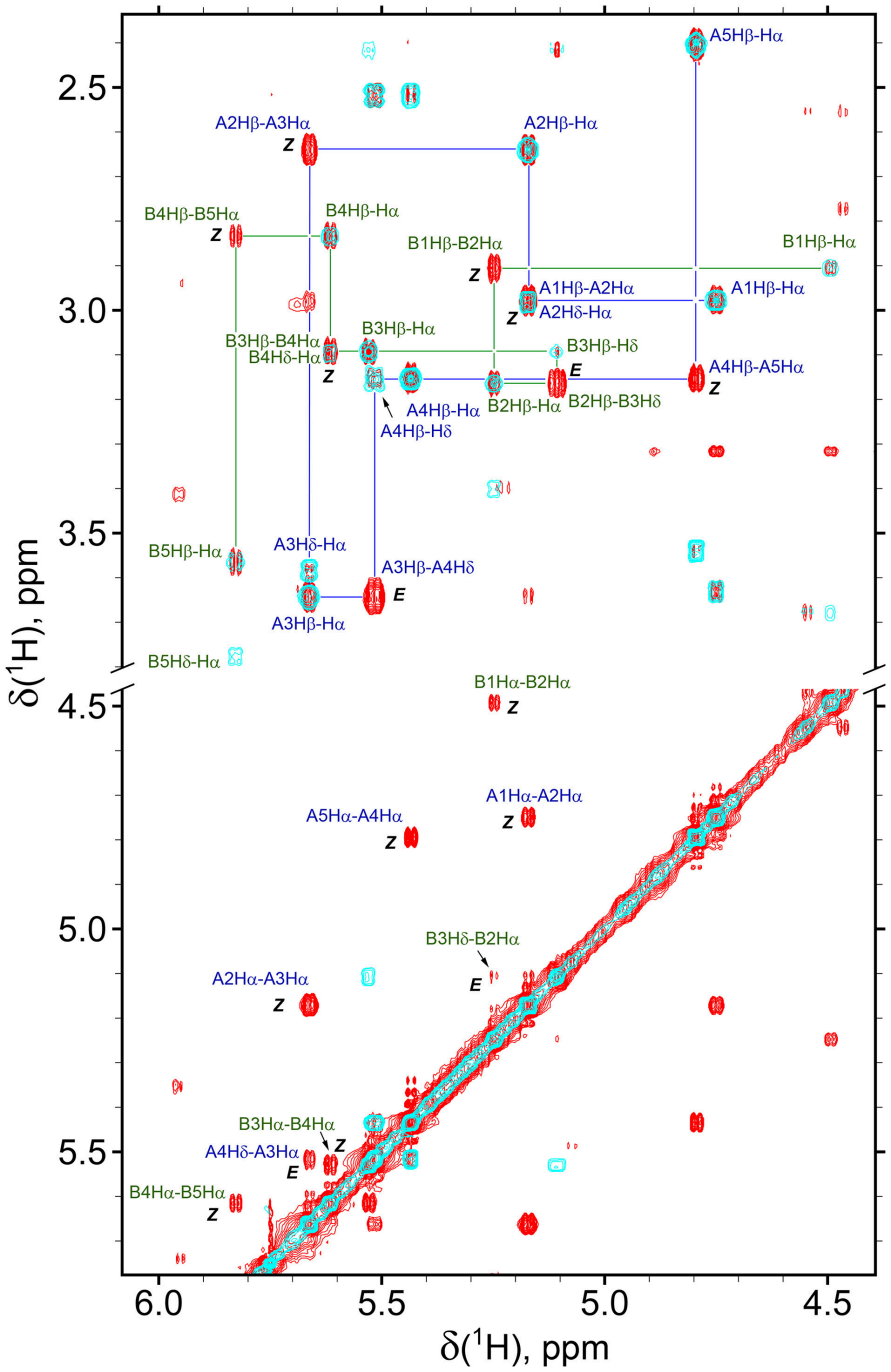
Overlay of the fragments of 2D NOESY (350 ms mixing time, red) and TOCSY (70 ms mixing time, cyan) spectra of β-proline pentapeptide **2** in DMSO solution. Shown are assignments of the resonances of two major conformers (A, *ZZEZ*; B, *ZEZZ*). Labeled are key NOEs characteristic for *Z* and *E* peptide bond configurations.

The observed isomerism for alternating β-proline oligomers is primarily determined by *cis-trans* orientation of the β-peptide bonds. *Trans* configuration correspond to *trans* orientation of Cα and O atoms around the N-C(O) peptide bond with the value of torsion angle Cβ^i^-C(O)^i^-N^i+1^-Cα^i+1^ closed to zero. *Trans-*configuration (*Z*) of the β-peptide bond is characterized by intense NOE (ROE) cross-peaks between Hβ_i_ and Hα_i+1_ protons (Figure 3A), while *cis*-configuration (*E*) is characterized by intense NOEs between Hβ_i_ and Hδ_i+1_ protons (Figure 3B). These characteristic NOEs (Figure 4) allow identifying uniquely the conformation of the polypeptide chain.

The observed conformational isomers of β-proline oligopeptides have different populations with (*ZZZ*)-configuration of the β-polyproline framework being a dominant conformer for the tetrapeptide and (*ZZEZ*)-configuration of the β-polyproline framework as a prevailing one for the pentapeptide (Table 1). The equilibrium between conformers shown in Figures 1, 2 is confirmed experimentally by the existence of exchange cross-peaks between the signals from different conformers observed in ROESY spectra (Supplementary Figure 1). Under equilibrium conditions, the relative energy of formation of each conformer can be derived from population values P_i_ using the Boltzmann equation ΔG_ij_ = -RTln(P_j_/P_i_). It can be seen that the *Z*↔*E* equilibrium is characterized by a 0.4–1.3 kcal·mol^−1^ energy difference (Table 1).

**Table 1:** Populations of conformations observed for β-proline tetrapeptide (**1**) and pentapeptide (**2**).

**Conformation**	**Population (%)**	**Relative energy of formation, kcal·mol^−1^**
**TETRAMER (1)**		
*ZZZ*	42	0
*ZZE*	23	0.36
*ZEZ*	16	0.56
*EZZ*	4	1.30
**PENTAMER (2)**		
*ZZEZ*	55	0
*ZEZZ*	21	0.57

**Figure 5:**
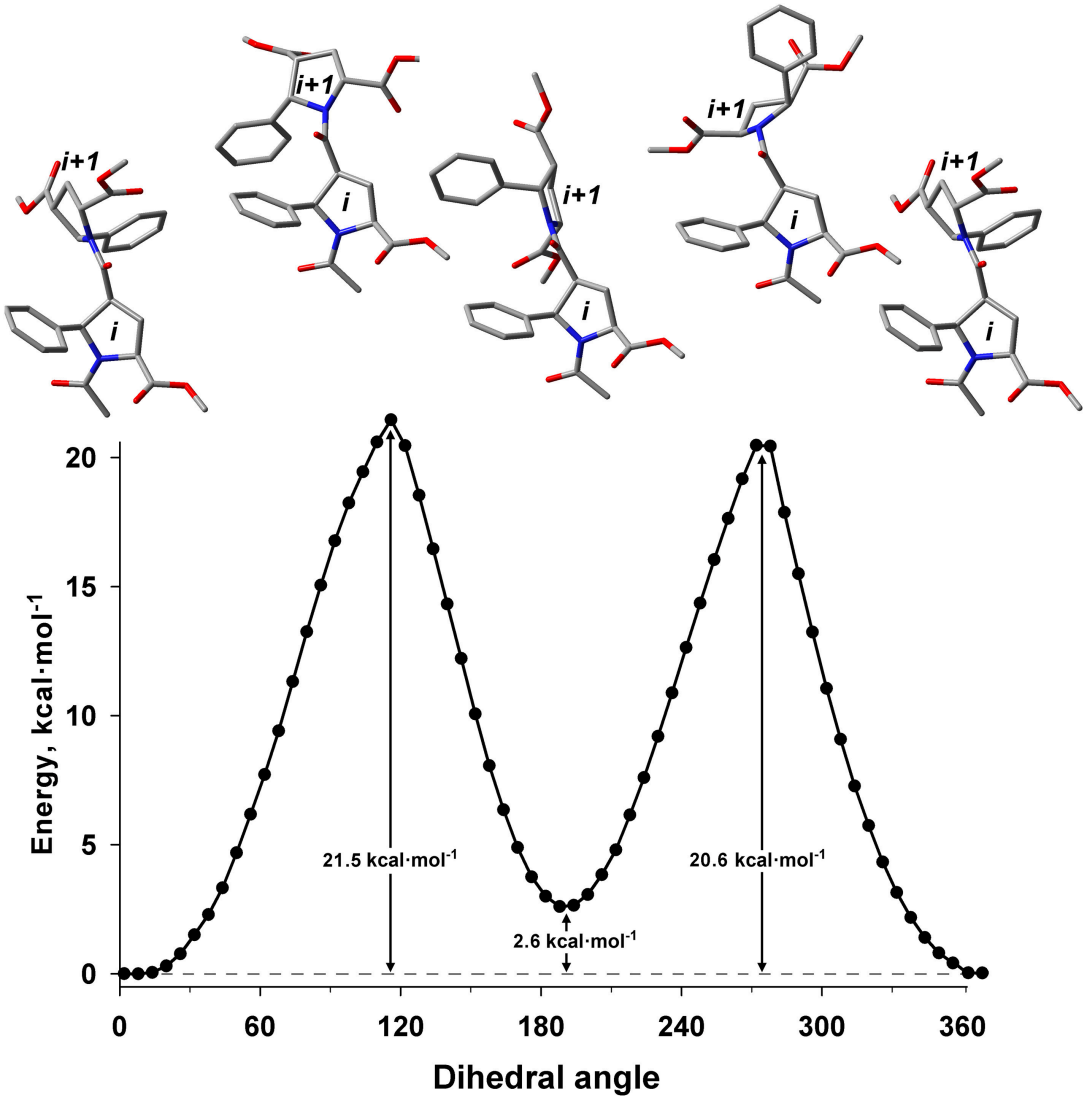
Energy profile for the transition between *Z* (ψ = 8°) and *E* (ψ = 191°) isomers in the alternating β-proline dimer obtained by DFT calculations using B3LYP at 6-311+G(d,p) in dimethyl sulfoxide environment with geometry optimization of intermediate states. Representative conformations are shown.

The energy difference and barrier between *Z* and *E* isomers of an alternating β-proline dimer were estimated using quantum mechanical calculations. Geometries of an alternating β-proline dimeric fragment corresponding to *Z* and *E* junctions of the residues were optimized with DFT calculations (Figure 3). The calculated difference in energy of formation between *Z* and *E* isomers is about 2.6 kcal·mol^−1^ (Figure 5), which is noticeably higher than the difference measured experimentally for β-proline tetra- and pentapeptides (Table 1). In the crystalline forms of similar alternating β-polyprolines we observed definite n → π^*^ interactions between the β-peptide carbonyl group and backbone ester functionality (Kudryavtsev et al., 2015a). Each of these interactions could contribute up to 0.8 kcal·mol^−1^ into the total energy decrease (Choudhary et al., 2009; Kubyshkin et al., 2016). Two or three cumulative n → π^*^ strengthening may explain discussed difference in the calculated value for the dimer and the observed magnitude for the oligomers in solution. Reciprocal carbonyl-carbonyl interactions recently discovered in polyproline II (PPII) helices (Rahim et al., 2017) may also be factor of the stabilization of *E* isomers.

The calculated energy barier ΔG‡ for the transition between *Z* and *E* isomers is 20.6 kcal·mol^−1^ (Figure 5). This is in good agreement with the value of approximately 19 kcal·mol^−1^ calculated from the analysis of the ^1^H line shapes of tetramer **1** *tert*-butyl signals in NMR spectra recorded in the temperature range between 298 and 408 K (Supplementary Figure 5). This value is also in good aggreement with ΔG measured for Z↔E transition in acetyl-L-proline-NHMe (20.4 kcal·mol^−1^) (Doshi and Hamelberg, 2009), acetyl-L-proline-OMe (21.1 kcal·mol^−1^) (Renner et al., 2001) and in nitrogen-pyramidalized bicyclic β-proline oligomers (17.8 kcal·mol^−1^) (Otani et al., 2017).

#### Pyrrolidine ring conformation

**Figure 6:**
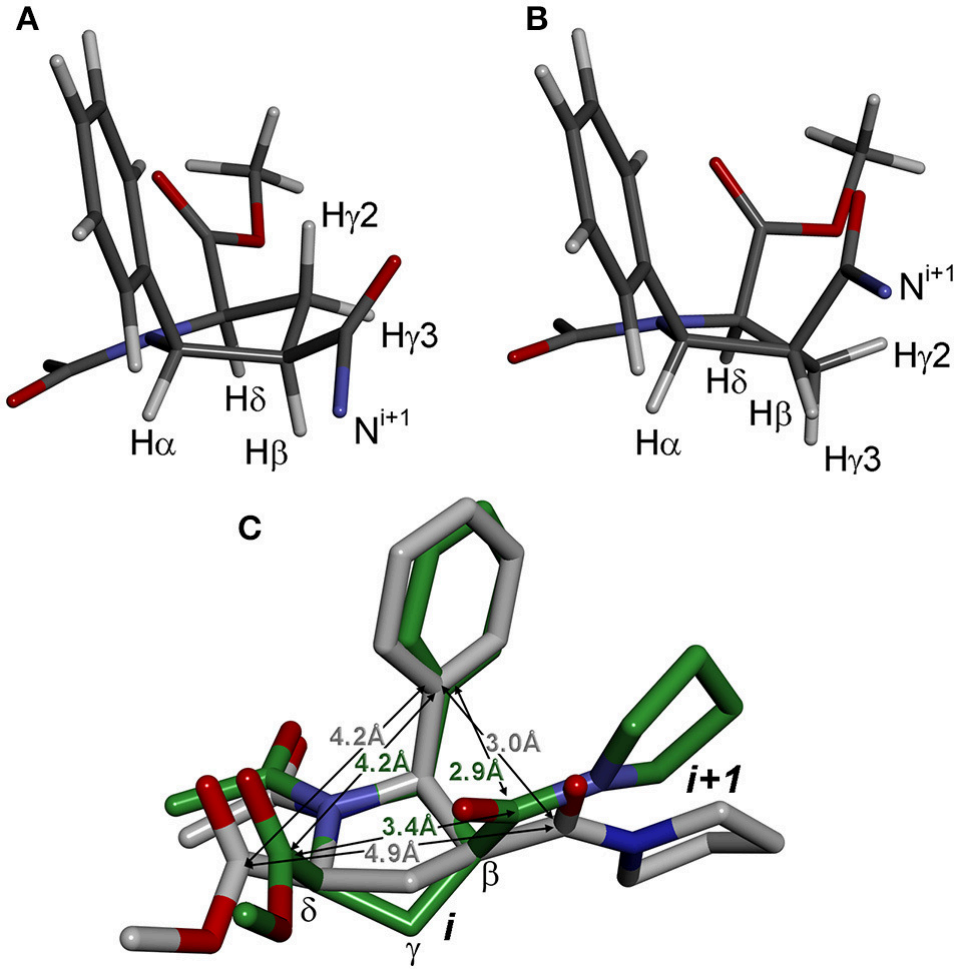
Conformation of β-proline ring: C^γ^-endo **(A)** and C^γ^-exo **(B)** puckers. C^γ^-endo is more stable than C^γ^-exo conformer with energy difference of 2.8 kcal·mol^−1^ in the gas phase and 1.2 kcal·mol^−1^ in dimethyl sulfoxide solution. **(C)** Overlay of C^γ^-endo (gray) and C^γ^-exo (green) puckers. Shown are distances between substituents in β-proline ring in two puckers.

The second source of structural flexibility of alternating β-proline oligopeptides is conformation of the pyrrolidine ring of 5-phenylpyrrolidine-2-carboxylate units. It can adopt either C^γ^-endo or C^γ^-exo conformations (Figure 6), similarly to that observed in N-acylated α-proline derivatives (DeRider et al., 2002). Quantum mechanical calculations determine that a C^γ^-endo pucker (Figure 6A) is more stable than C^γ^-exo state (Figure 6B). The difference in the energy of formation between endo- and exo-forms is 1.2 kcal·mol^−1^ in dimethyl sulfoxide solution and 2.8 kcal·mol^−1^ in the gas phase. The substituents in β and δ positions of the β-proline ring are spatially close in the C^γ^-exo pucker (Figure 6C), which restricts the rotation around the amide and C(O)-Cβ bonds connecting the two β-proline residues in this conformation (see below).

The C^γ^-endo and exo conformers have characteristic patterns of coupling constant values (see Table 2), which allows identification of these conformers experimentally. The C^γ^-exo pucker is characterized by the large differences in the values of coupling constants that involve Hγ2 and Hγ3 protons, whereas in C^γ^-endo conformation all vicinal coupling constants have a similar value. Experimental NMR data (Supplementary Figures 6, 7) indicate that all the 5-phenylpyrrolidine-2-carboxylate residues in the alternating β-proline tetrapeptide **1** and pentapeptide **2** exist in a C^γ^-endo conformation. All β-proline residues of the tetrapeptide (*ZZE*) conformer of the alternating β-proline tetrapeptide **1** are in C^γ^-endo conformation in the crystal structure (Kudryavtsev et al., 2015b).

**Table 2:** Calculated vicinal coupling constants (Hz) for C^γ^-endo and C^γ^-exo states of 5-phenylpyrrolidine-2-carboxylate unit.

**Coupling constant**	**C^γ^-endo pucker**	**C^γ^-exo pucker**
^3^J(Hα,Hβ)	8.7	9.6
^3^J(Hβ,Hγ2)	11.6	0.5
^3^J(Hβ,Hγ3)	6.1	9.0
^3^J(Hδ,Hγ2)	9.7	0.7
^3^J(Hδ,Hγ3)	6.9	10.0

#### Rotation around Ψ main-chain bond

**Figure 7:**
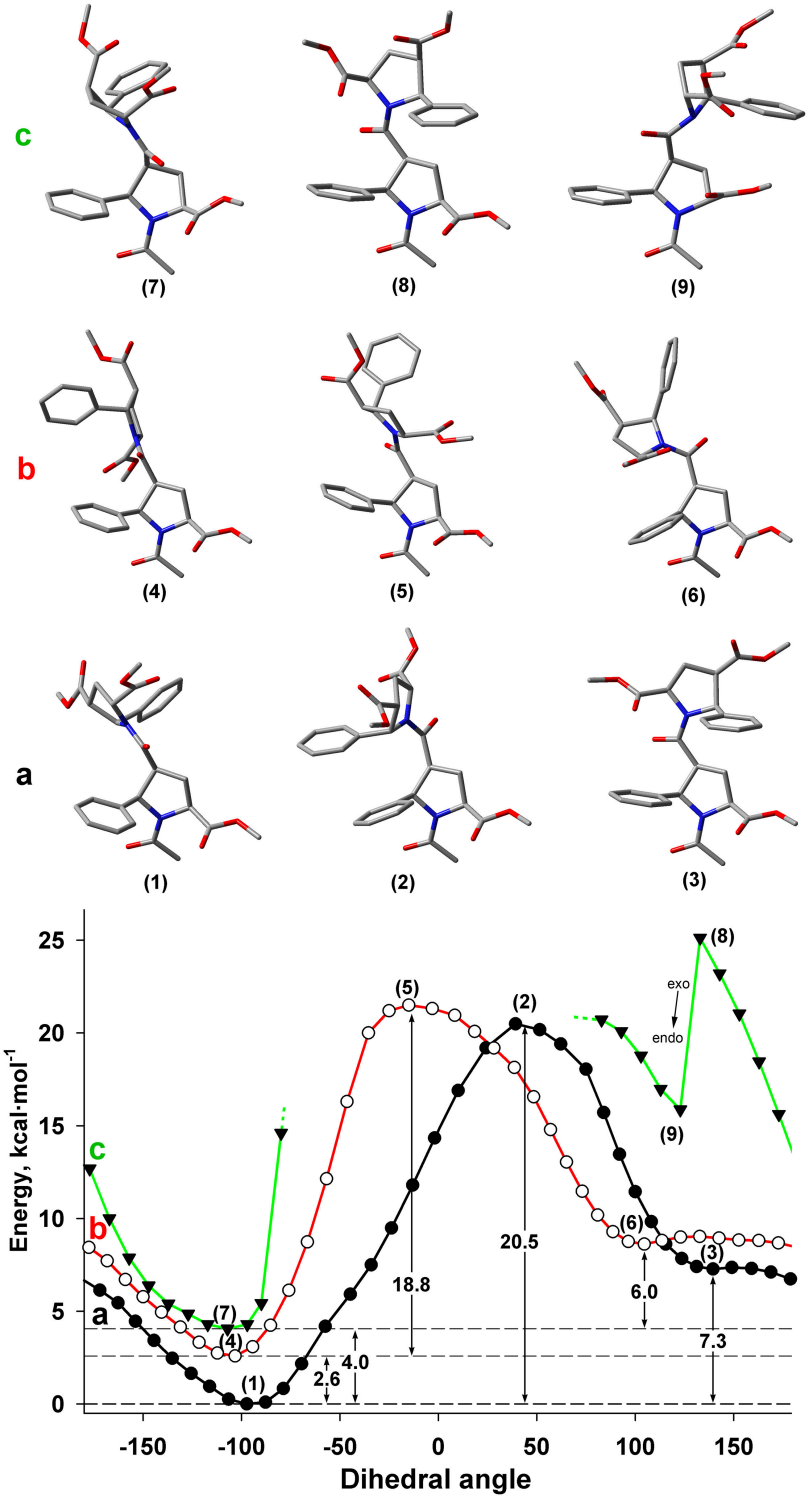
Energy profile for the rotation around dihedral angle ψ (N-C(O)-Cβ-Cα) for *Z* and *E* isomers of alternating β-proline dimer obtained by DFT calculations with B3LYP at 6-311+G(d,p) in dimethyl sulfoxide environment with geometry optimization of intermediate states. **(a)** Calculations, performed for *Z* isomer of C^γ^-endo, C^γ^-endo dipeptide (black line, filed circlers). **(b)** Calculations, performed for *E* isomer of C^γ^-endo, C^γ^-endo dipeptide (red line, open circles). **(c)** Calculations, performed for *Z* isomer of C^γ^-exo, C^γ^-exo dipeptide (green line, triangles). Representative conformations are shown. C^γ^-endo conformation of β-proline ring is stable (points 1–6), whereas C^γ^-exo conformation (points 7 and 9) is switched to C^γ^-endo conformation (point 8) in attempt to minimize steric clashes.

Another possible source of conformational diversity of the studied β-proline oligopeptides is rotation around bond Cβ-C(O), which corresponds to the dihedral angle ψ of polypeptide chain (Cα^i^-Cβ^i^- C(O)^i^-N^i+1^). Figure 7 shows the energy profile of such rotation for both *Z* and *E* conformers of the dipeptide fragment in DMSO solution.

It can be seen that both *Z* and *E* isomers have the only one dominant conformation of energy minima with the value of ψ dihedral angle of −77 and −103° correspondingly (Figures 7A,B). Alternative conformations with 130 and 105° for *Z* and *E* isomers have much higher energy. The energy of the transition state between these two minima corresponds to similar value of the energy barrier between *Z* and *E* isomers (Figure 5).

The steric effects involving a bulky phenyl ring seemingly determine the existence of a single dominant conformation. Thus, the highest energy barrier observed in the *Z* isomer is due to the steric interaction of the two phenyl rings from the neighboring residues (Figure 7). The steric effect became decisive for the C^γ^-exo puckering of pyrrolidine ring. The spatial proximity of the groups in β and δ positions of the β-proline ring in C^γ^-exo state impedes rotation around the ψ dihedral angle. Such rotation becomes possible only upon transition of the β-proline ring to the C^γ^-endo conformation (Figure 7c). Quantum mechanical energy scanning calculations do not imply dynamic processes. In reality, to rotate around ψ dihedral angle, pyrrolidine ring should first switch its conformation to C^γ^-endo state.

### Structure of β-proline oligopeptides in solution

**Figure 8:**
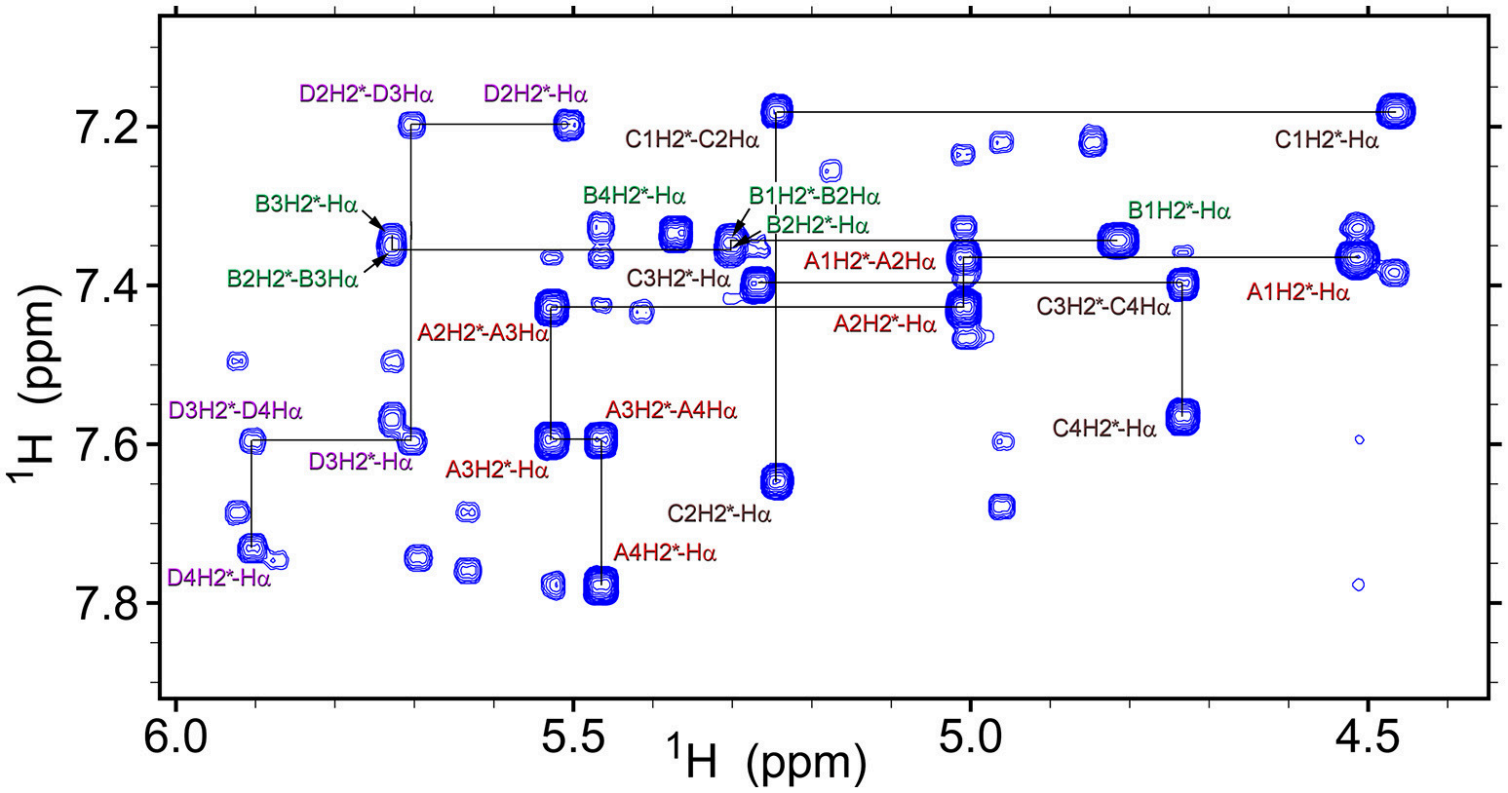
A fragment of the ROESY spectrum (300 ms mixing time) of tetrapeptide (**1**) recorded at 298K in DMSO-d_6_. Conformers assignments are: A, *ZZZ*; B, *ZZE*; C, *ZEZ*; D, *EZZ*. Several representative intra-residual and sequential NOEs are marked.

Analysis of NOESY and ROESY spectra (Figures 4, 8, Supplementary Figure 1) of β-proline oligopeptides **1** and **2** allowed collecting set of distance restraints sufficient to calculate structures of all the observed conformers mentioned above (Table 1). Structural calculations were performed by the restraint molecular dynamics using the simulated annealing protocol.

**Figure 9:**
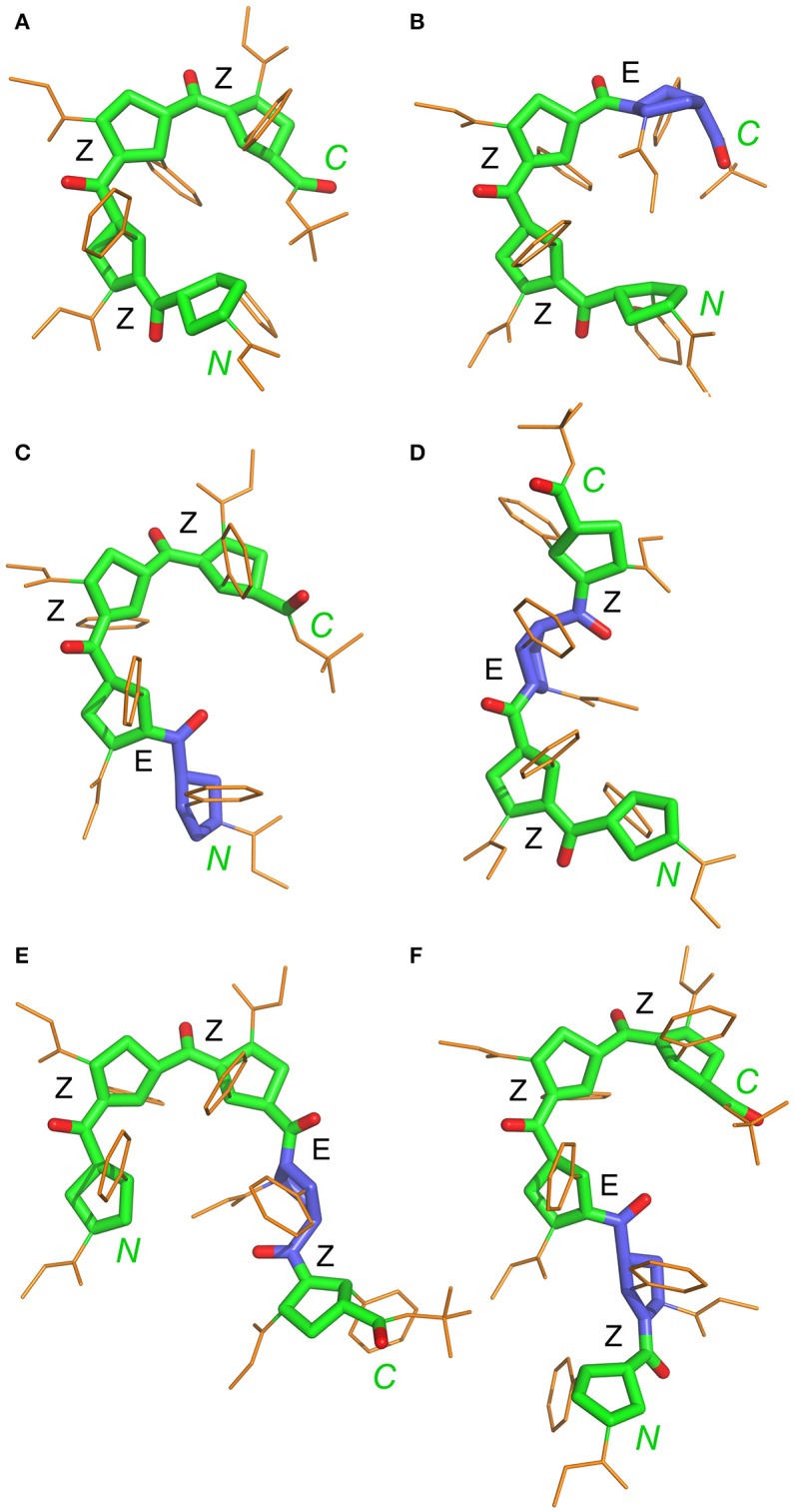
Representative NMR structures of the alternating β-proline tetramer **1 (A–D)** and the alternating β-proline pentamer **2 (E,F)**. β-Peptide frameworks are shown by thicker sticks, side chains shown by thin gold sticks. Labeled are N and C-terminal residues. Configuration of each β-peptide bond is marked (*Z* or *E*). Residues with *E*-configuration of β-peptide bond are highlighted by purple color.

Statistical evaluations for the ensembles of calculated NMR structures (Supplementary Figure 2) are presented in Table 3. The total number of NOEs used for structural calculations are between 9 and 19 per pyrrolidine residue. An ensemble of the 20 best-energy NMR models was calculated for each conformer. Representative structures of each conformer of β-proline oligopeptides are shown in Figure 9. Supplementary Figure 8 shows distribution of the dihedral angles that represent the pyrrolidine ring conformation (Supplementary Figure 8A), ψ main-chain bond (Supplementary Figure 8B), and amide bond (Supplementary Figure 8C). The largest distribution is observed for the ψ bond, whereas pyrrolidine ring puckering and angles that represent *Z* and *E* configurations vary in narrow ranges. The backbone of the dominant (*ZZZ*)-tetramer **1** forms a right-handed coil with a weak propensity toward a helical conformation (Figure 9A). The methoxycarbonyl side chains are oriented radially outward the coil trace, whereas phenyl rings are situated perpendicular to the coil plane. Due to the alternation of *RRS* and *SSR* stereogenic centers in pyrrolidine units the phenyl rings of the neighboring residues having the *Z*-configuration of the amide bond oriented in opposite directions to the coil plane. *E*-configuration of the β-peptide bond makes orientation of the neighboring phenyl groups occur at the same side of the virtual plane.

**Table 3:** Statistics for the ensembles of the calculated NMR structures of four conformations of β-proline tetrapeptide (**1**) and two conformations of β-proline pentapeptide (**2**).

	**Tetrapeptide (1)**				**Pentapeptide (2)**	
	***ZZZ***	***ZZE***	***ZEZ***	***EZZ***	***ZZEZ***	***ZEZZ***
Structures in NMR family	20	20	20	20	20	20
Total number of NOEs	75	52	54	34	65	46
Intraresidue	34	36	34	24	35	27
Sequential	32	12	15	9	29	19
Medium-range^a^	9	4	5	1	1	0
Number of NOE violations (>0.3 Å) per structure	0	0	0	0	0	0
RMSD^b^ (Å)	0.11	0.13	0.06	0.04	0.35	0.17

a NOEs between residues i and j, where 1 < |i–j| ≤ 4.

b *RMSD of coordinates of atoms C', Cα, Cβ, and N for superposition of the family of structures over the representative structure in the family*.

Conformation of representative NMR structure for each conformer was optimized using quantum mechanics with B3LYP/6-31+G(d,p) level for tetramer **1** and with HF/6-31G(d,p) level for pentamer **2**. Overall conformation of NMR and QM structures were similar (Supplementary Figures 3, 4), with RMSD for heavy main chain atoms N, Cα, Cβ, Cγ, Cδ, C, and O of all β-proline rings in the range between 0.40 and 1.0 Å. The calculated structure of (*ZZE*)-conformer of the tetramer **1** resembled the solid state structure determined by X-ray analysis (Kudryavtsev et al., 2015b). The X-ray structure of the (*ZZE*)-conformer of tetramer **1** differs from the NMR determined structure and its DFT optimized structure with RMSD 0.86Å and 0.77Å respectively (Supplementary Figure 3).

N-terminal and C-terminal residues in (*ZZZ*)-conformer of tetrapeptide **1** are in close spatial proximity. There are strong NOEs between protons from *tert*-butyl group of the C-terminal residue and the α-phenyl ring at the N-terminal residue. Such NOEs are not observed for other conformers of tetrapeptide **1**.

Extension of this tetrapeptide by an additional residue with *Z*-orientation of its β-peptide bond would cause steric clashes. Therefore, *E*-orientation in at least one β-peptide bond is an obligatory condition for the stability of discussed β-proline oligomers with a number of pyrrolidine residues larger than 4. Thus, pentapeptide **2** exists in solution in two main conformational states, (*ZZEZ*) and (*ZEZZ*) (see Figure 9 and Table 1). It should be noted that equilibrium between conformers (*ZZEZ*) and (*ZEZZ*) and the absence of the (*ZZZZ*) conformer indicate a synchronous change of the configuration of three peptide bonds. This distinguishes the investigated β-peptides from their α-proline analogs, for which transition from *E* to *Z* configurations occurs in a cascade fashion (Shi et al., 2014).

**Figure 10:**
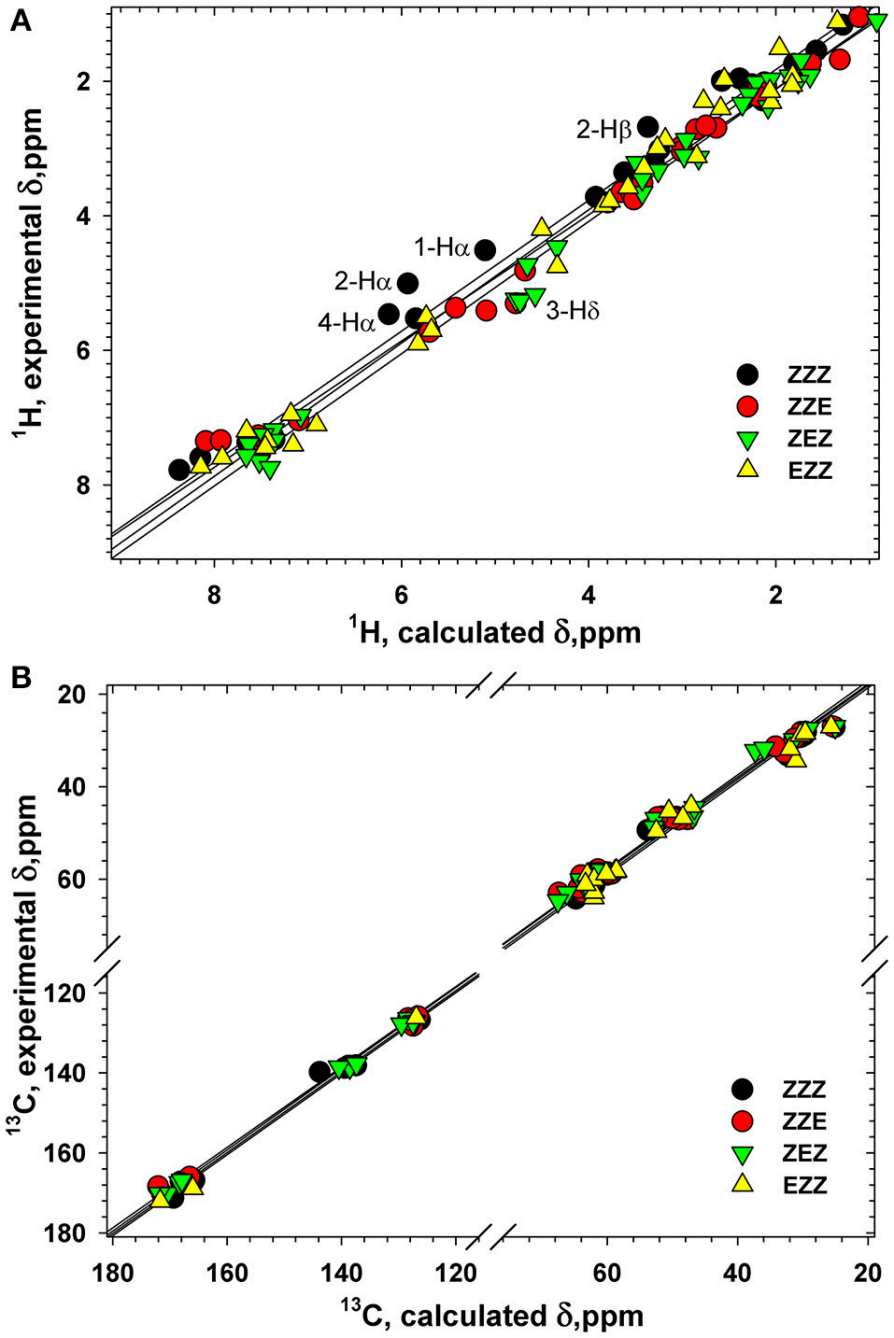
Correlation between the calculated and experimental ^1^H **(A)** and ^13^C **(B)** chemical shifts (ppm) in four conformers of tetrapeptide **1**. Labeled are the atoms with the largest differences between the calculated and experimental ^1^H chemical shifts.

Various orientation of the side chains, particularly phenyl rings, lead to a large dispersion of the chemical shifts of the β-proline oligopeptides **1** and **2** (Kudryavtsev et al., 2015b). Thus, ^13^C and ^1^H chemical shifts can be considered as informative parameters which characterize the correct folding of the β-proline oligopeptide chain. Figure 10 shows correlation between the experimentally measured and computed by DFT ^1^H and ^13^C chemical shifts of tetramer **1** (Tables S1–S4). The Pearson correlation coefficient *R*^2^ for ^1^H chemical shifts is 0.989 and for ^13^C chemical shifts is 0.999. There are several nuclei whose resonance are distinctive for the peptide conformation. Thus, a change of the peptide bond configuration from *Z* to *E* causes a 1–2 ppm low field shift of the Hδ proton and a 2–3 ppm high field shift of Cβ carbon-13.

It can be observed that a tripeptide fragment with (*ZZ*)-configuration of two β-peptide bonds exists in almost all the structures shown in Figure 9. Interestingly, the elongation of the polypeptide chain is accompanied by a decrease in the conformational diversity of the molecule (four major conformers in tetramer and only two in pentamer). This can be explained by the narrow corridor in the conformational space due to significant steric hindrances. Experimentally obtained structures of the tetramer **1** and pentamer **2** conformers and determined ranges of peptide bond and ψ angles allowed to model conformation of larger alternating β-proline oligopeptides. Supplementary Figure 9 shows the most probable conformation of an alternating β-proline octapeptide. It was build by superposition of (*ZZE*) and (*ZEZZ*) structures. Only such combination allowed avoiding steric clashes. It can be proposed that the most probable configuration of the β-proline polypeptide should contain *ZZ* fragments interleaved with *EZE* motifs. The molecular structure of the longer β-proline peptide consisting of 21 monomeric units which form (*ZZ*)*-* and (*EZE*)*-*repetitive motifs is represented on Supplementary Figure 10.

Different orientations of the side chains from adjacent residues provide a rich diversity of overall shapes of the studied molecules **1** and **2**. The existence of molecules in several structural diverse conformations with a limited number of such conformational states, makes the studied β-proline oligopeptides promising compounds capable to bind their potential biological targets by choosing the optimal conformational state for binding.

## Conclusions

Three major factors that control the structural diversity of the studied alternating β-proline oligopeptides are the conformation of the pyrrolidine ring, the *Z*/*E* isomerism of β-peptide bonds and hindered rotation of the neighboring monomers around the dihedral angle ψ (N-C(O)-Cβ-Cα). In all three cases, the energy of the dominant state is significantly lower than the alternative variants, which predetermines a significant narrowing of the structural diversity of the alternating β-proline oligopeptides. The energy difference between *Z-* and *E*-β-peptide bond isomers turned out to be lower than would be theoretically predicted, which makes the existence of an *E*-β-peptide bond possible for at least one residue. For oligopeptides with a chain length greater than 4, the presence of *E* configuration is mandatory, since a chain of four monomers divided by three *Z*-β-peptide bonds forms a pseudo-cycle where the first and fourth residues are close to each other, and the additional *Z*-orientation is not feasible due to steric problems. It is worth noting that concerted Z↔E backbone isomerization is observed for the alternating β-proline pentapeptide with a synchronous change of the configurations of three peptide bonds. This distinguishes the studied β-proline oligopeptides from their α-analogs where polyproline backbone isomerization proceeds in a cascade fashion from one terminus to another. Modeling predicts an extended wave-like topology of an longer alternating β-proline polypeptide chain that contains interleaved *ZZ* and *EZE* fragments. Conversion between a limited number of alternating β-proline tetramer and pentamer conformers with a compatible energy of formation, but substantially different spatial organization, enlarges the diversity of molecular shapes. This property provides increased adaptability of binding of the studied β-proline oligopeptides to their potential biomacromolecular targets.

## Author contributions

SB, KK, and VP, designed the research and wrote the manuscript; OS: performed the NMR experiments; PI: synthesized the oligopeptides; AM: analyzed NMR spectra and performed structure calculations; VP: performed DFT calculations and analyzed the results.

### Conflict of interest statement

The authors declare that the research was conducted in the absence of any commercial or financial relationships that could be construed as a potential conflict of interest.
