# Supplementary material for: Theoretical and NMR Conformational Studies of β-Proline Oligopeptides With Alternating Chirality of Pyrrolidine Units
Source: Front Chem. 2018 Mar 28;6:91. doi: 10.3389/fchem.2018.00091 (PMC5883087; doi:10.3389/fchem.2018.00091)
Supplement: Supplementary file 1 [file Presentation1.PDF]

Supplementary Material

Theoretical and NMR conformational studies of β-proline oligopeptides with alternating chirality of pyrrolidine units

Alexey B. Mantsyzov, Oleg Yu. Savelyev, Polina M. Ivantcova, Stefan Bräse, Konstantin V. Kudryavtsev*, and Vladimir I. Polshakov*

*** Correspondence:** Vladimir Polshakov: vpolsha@mail.ru; Konstantin Kudryavtsev: kudr@med.chem.msu.ru

1 Supplementary Figures

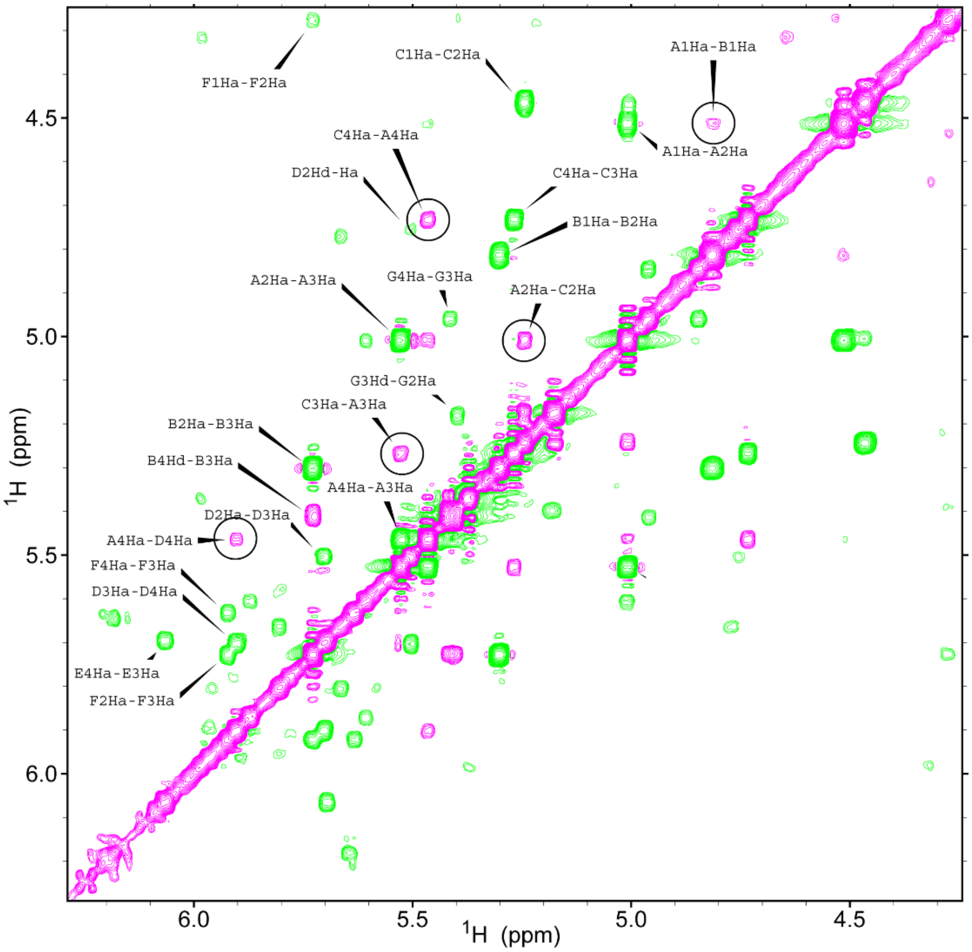

Supplementary Figure 1. Fragment of the ROESY spectrum (300 ms mixing time) of the alternating β-proline tetramer **1** recorded at 298K in DMSO-d_6. Shown are cross-peaks observed for the isomers *ZZZ* (A), *ZZE* (B), *ZEZ* (C) and *EZZ* (D). Cross-peaks with negative intensities (shown by green) correspond mainly to the through-space cross-relaxation (NOE). Cross-peaks with positive intensities (shown by magenta) originate mainly from the chemical exchange between the conformers. Circled are exchange cross-peaks.

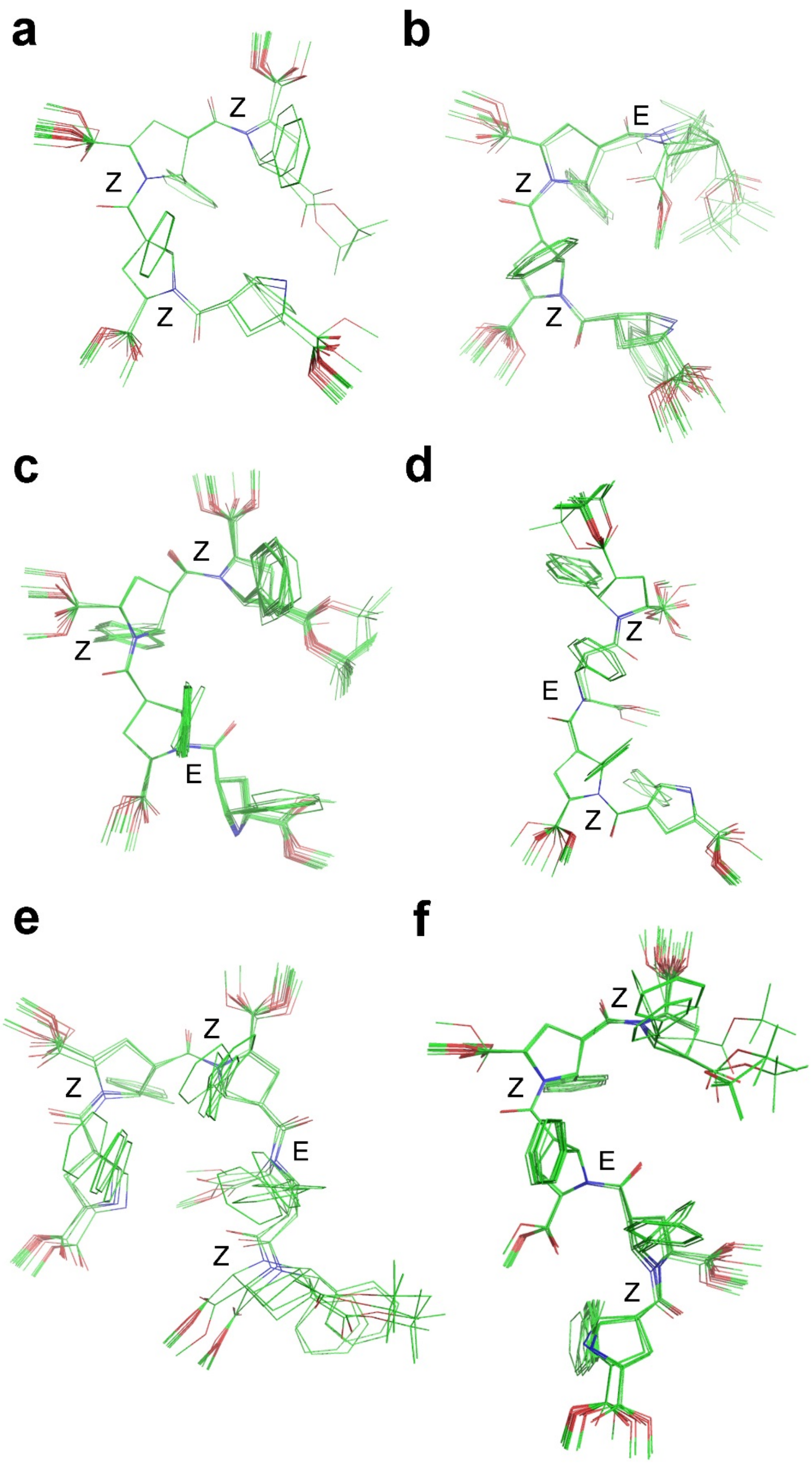

Supplementary Figure 2. Families of 20 NMR structures of the alternating β-proline tetramer 1 (a-d) and the alternating β-proline pentamer 2 (e, f).

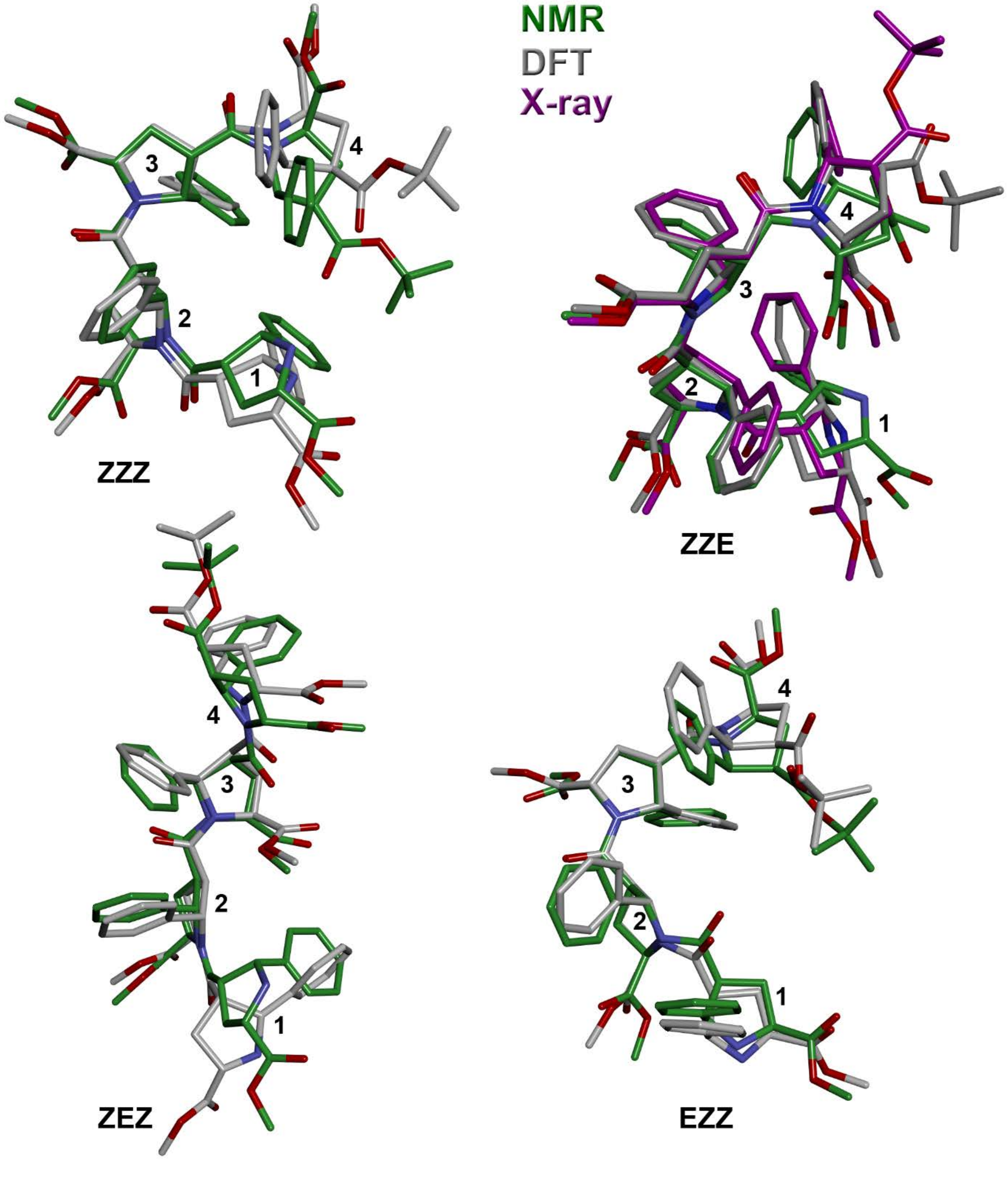

Supplementary Figure 3. NMR and DFT optimized structures of four conformations of the alternating β-proline tetramer **1**. Shown is also crystallographic structure of the ZZE conformer. All structures were superimposed using the set of heavy main chain atoms of four β-proline residues and three peptide bonds (N, Cα, Cβ, Cγ, Cδ, C and O). Values of pairwise RMSD after superposition of NMR on DFT structures are 0.68Å for ZZZ, 0.40Å for ZZE, 1.04Å for ZEZ and 0.46Å for EZZ conformers. RMSD after superposition of ZZE NMR structure on crystallographic structure is 0.86Å, and DFT on X-ray structure is 0.77Å.

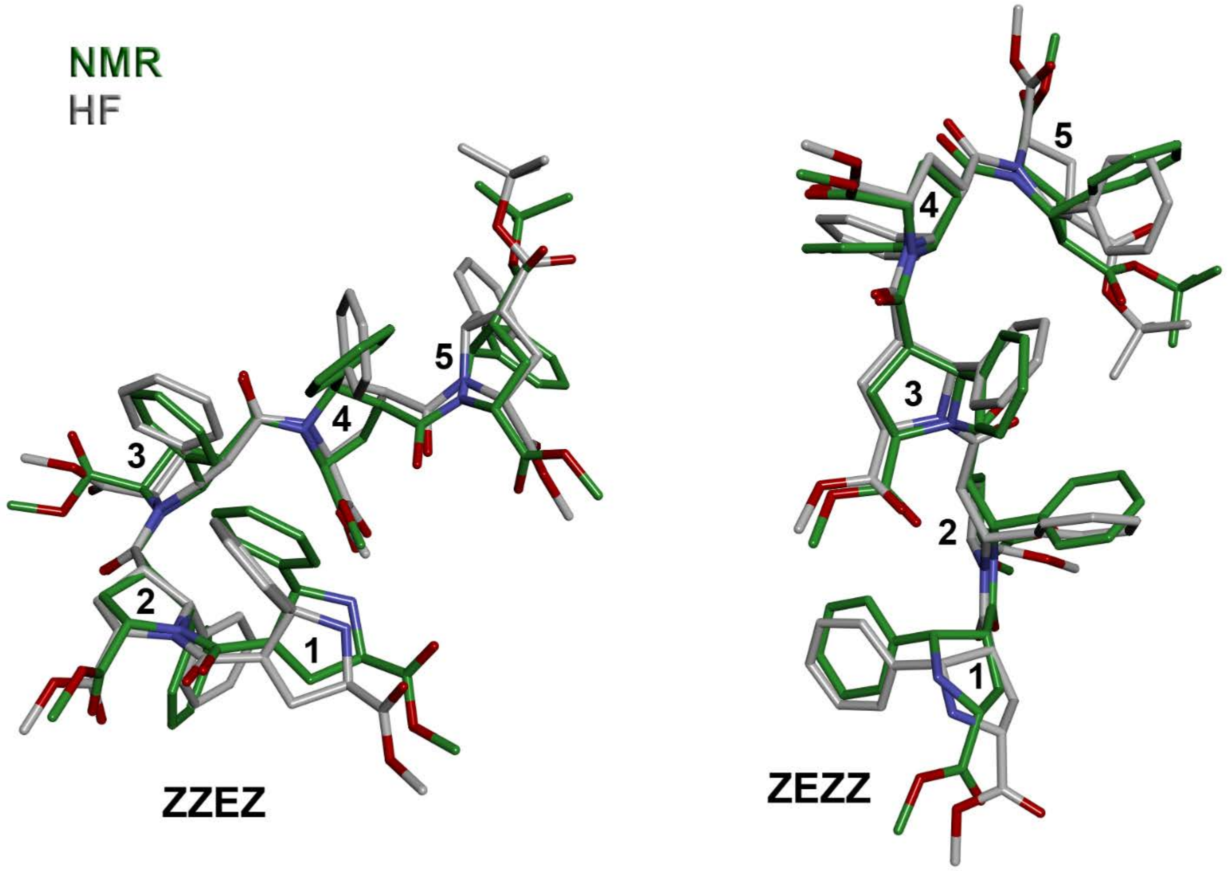

Supplementary Figure 4. NMR and HF optimized structures of two conformations of the alternating β-proline pentamer **2**. Structures were superimposed using the set of heavy main chain atoms of five β-proline residues and four peptide bonds (N, Cα, Cβ, Cγ, Cδ, C and O). Values of pairwise RMSD after superposition of NMR on HF structures are 0.50Å for ZZEZ and 0.52Å for ZEZZ conformers.

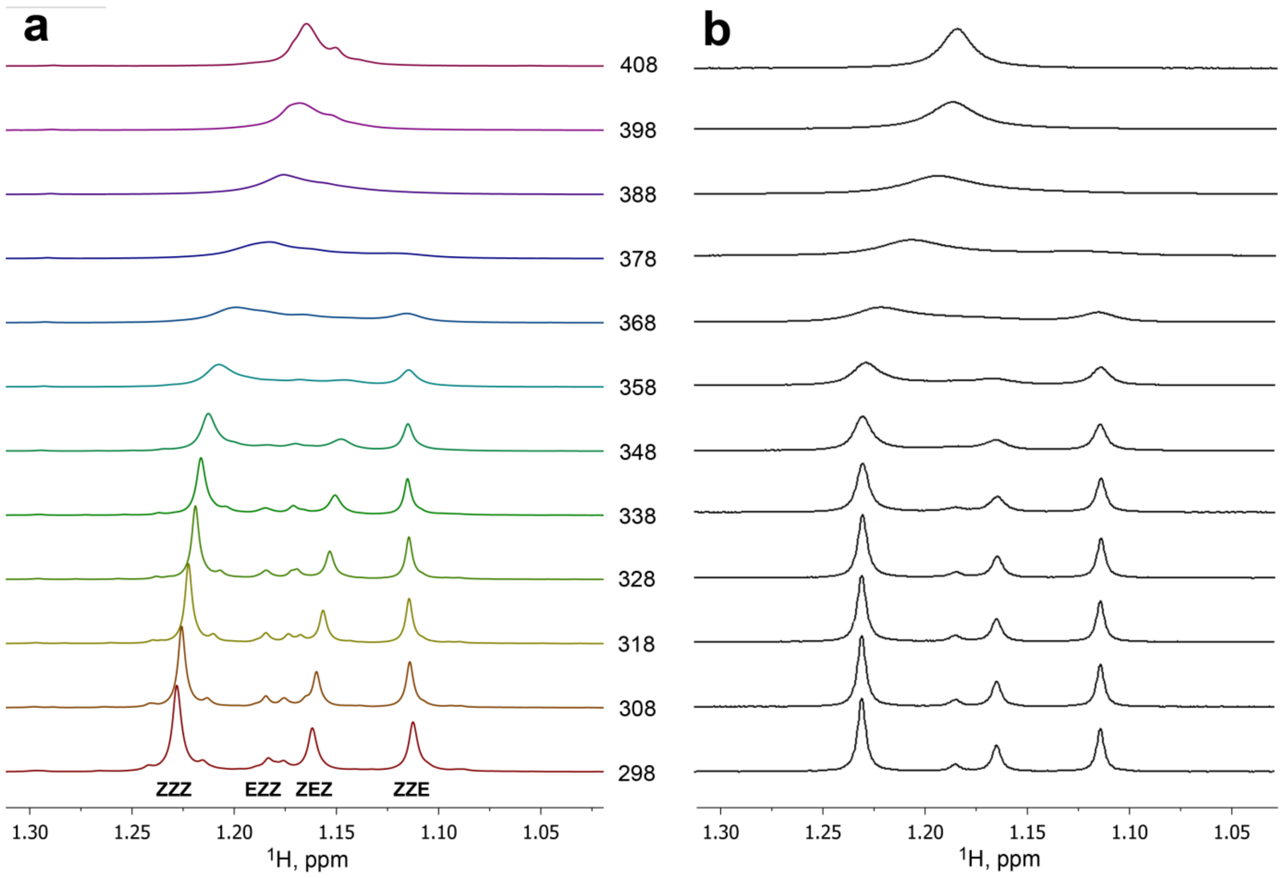

Supplementary Figure 5. Fragments (1.0 – 1.3 ppm) of ^1H NMR spectra of tetramer with the resonances from t-Bu groups of the C-terminal β-proline residues. (a) Experimental spectra recorded in DMSO-d6 the temperature range between 298 and 408K. (b) Calculated ^1H spectra of the four-site exchange system, which simulates behavior of the t-Bu groups of four major conformers of the tetramer. Multiple-site exchange simulation of the NMR spectra were carried out with the use of written-in-house program Muses (<u>Mu</u>ltiple-<u>s</u>ite <u>e</u>xchange <u>s</u>imulation). The following parameters were used for the calculations. Chemical shifts (in ppm) and populations (in brackets) of the ZZZ (A), ZZE (B), ZEZ (C) and EZZ (D) conformations are 1.225 (0.489), 1.110 (0.266), 1.160 (0.191) and 1.180 (0.054). Initial (natural) line width of each signal was set to 2.3 Hz. Components of exchange rate matrix (in s^{-1}) are A↔B 0.54, A↔C 0.78, A↔D 0.33. Direct exchange between sites B↔C, B↔D and C↔D were assumed to have zero exchange rate (i.e. such exchange does not exist). Each spectrum was calculated with the following multiplications of the exchange matrix: 1.0 (which corresponds to 298K of the experimental spectrum), 1.2 (308K), 2.0 (318K), 3.0 (328K), 5.9 (338K), 12.0 (348K), 20.0 (358K), 58.0 (368K), 198 (378K), 500 (388K), 1050 (398K) and 3000 (408K). Such change of the exchange rates correspond to the value of ~19 kcal·mol-1 for the $\Delta G^{\ddagger}$ of transition between Z and E isomers.

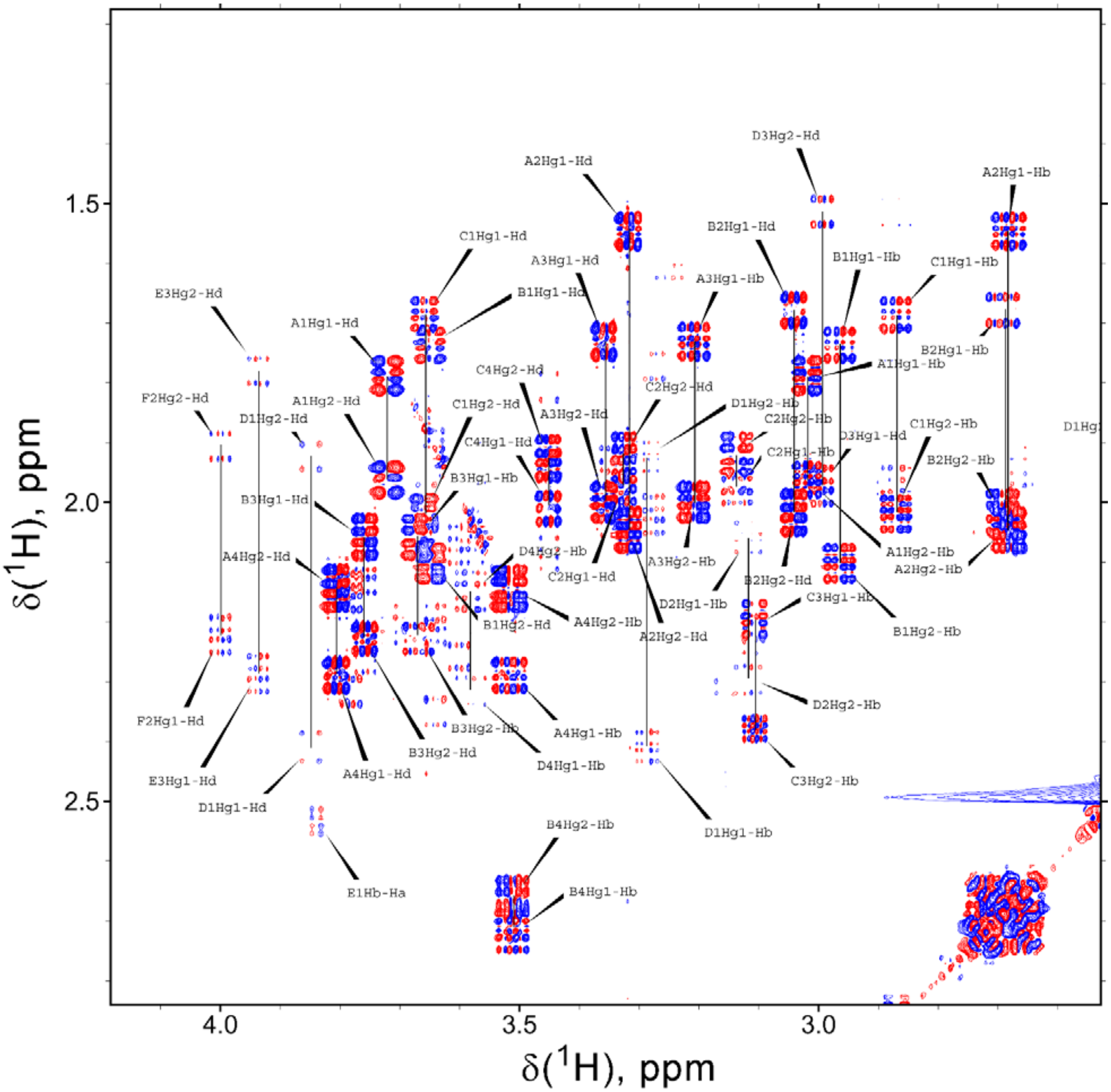

Supplementary Figure 6. Fragment of the DQF-COSY spectrum of the alternating β-proline tetramer 1 recorded at 298K in DMSO-d_6. Shown are cross-peaks observed for the isomers *ZZZ* (A), *ZZE* (B), *ZEZ* (C) and *EZZ* (D). Presence of correlations of both Hγ protons (Hγ1 and Hγ2) to Hβ and Hδ indicates C^{γ}-endo states of β-proline rings in all isomers. Minor components E and F correspond to the products of non-stereoselective cycloaddition with altered configurations of stereogenic centers.

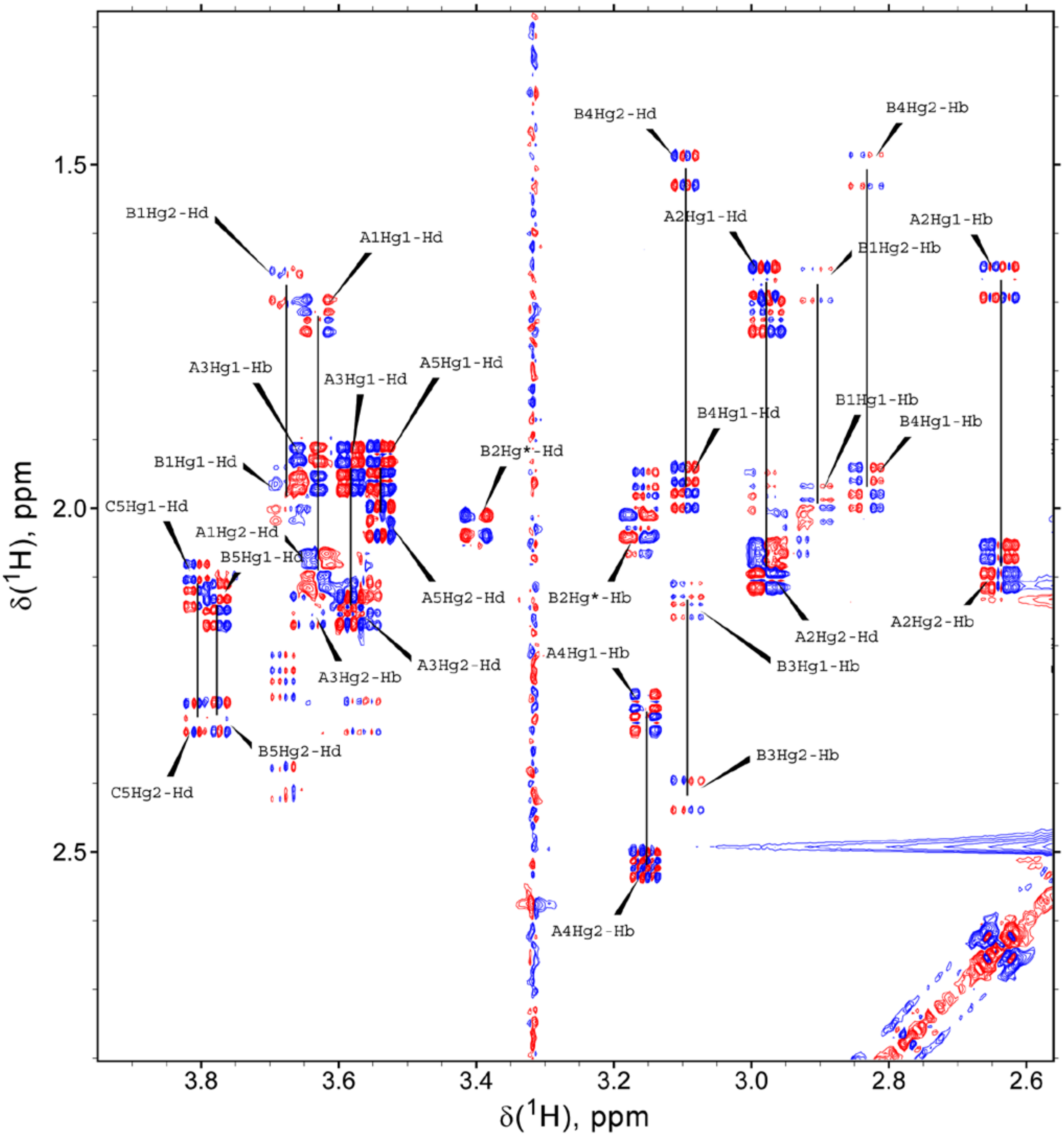

Supplementary Figure 7. Fragment of the DQF-COSY spectrum of the alternating β-proline pentamer 2 recorded at 298K in DMSO-d_6. Shown are cross-peaks observed for the isomers *ZZEZ* (A) and *ZEZZ* (B). Presence of correlations of both Hγ protons (Hγ1 and Hγ2) to Hβ and Hδ indicates C^{γ}-endo states of β-proline rings in both isomers. Minor component C corresponds to the product of non-stereoselective cycloaddition with altered configurations of stereogenic centers.

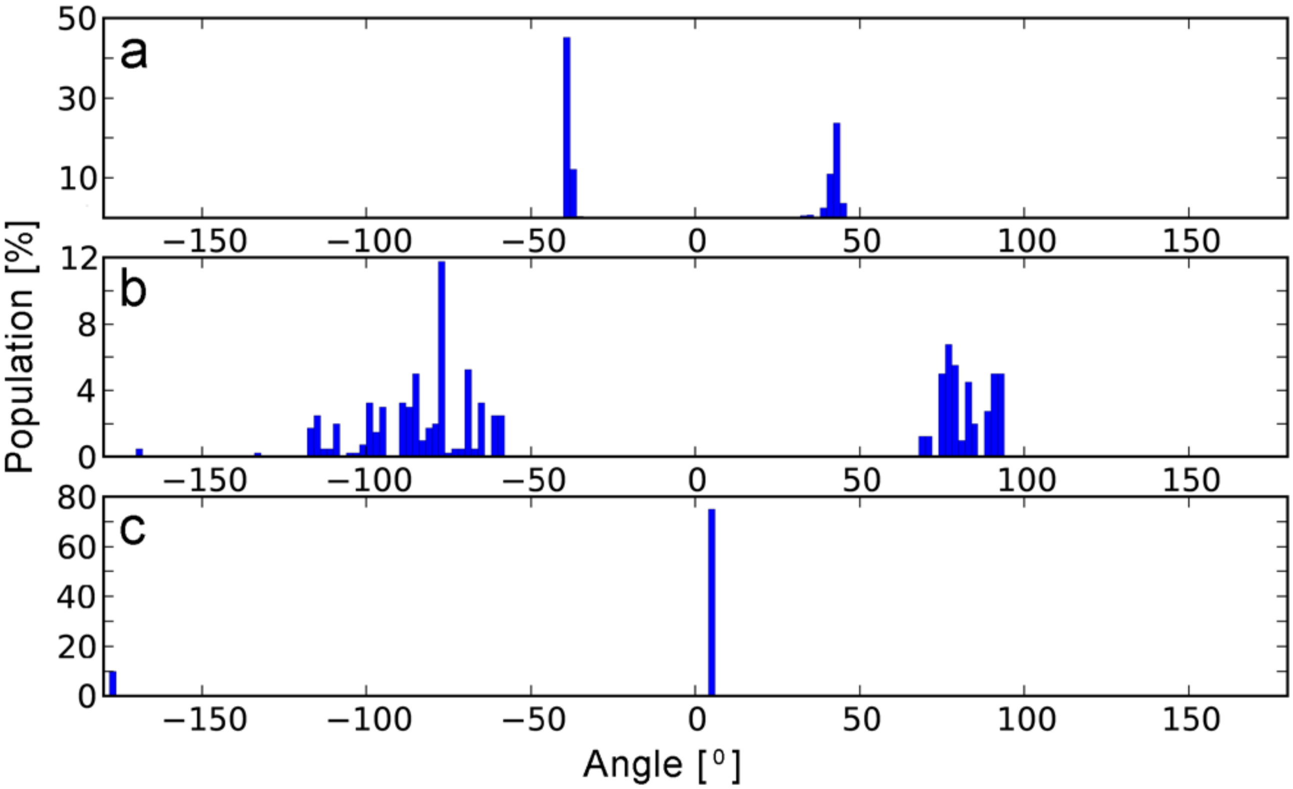

Supplementary Figure 8. Population distributions of the dihedral angles in the families of NMR structures of four conformers of tetrapeptides 1 and two conformers of pentapeptides 2. **A.** Dihedral angle $C\alpha^i$-$C\beta^i$-$C\gamma^i$-$C\delta^i$ that represents endo β-proline ring puckering. Two clusters correspond to alternative configurations of the β-proline rings: -40°±4° for Cα(S), Cβ(R), Cδ(R), and +40°±4° for Cα(R), Cβ(S), Cδ(S) **B.** ψ main-chain bond $C\alpha^i$-$C\beta^i$-$C(O)^i$-N^{i+1}. Two clusters of values -90°±30° and +85°±15° represent Cα(R), Cβ(S), Cδ(S) and Cα(S), Cβ(R), Cδ(R) configurations correspondingly. **C.** Peptide bond angle $C\beta^i$-$C(O)^i$-N^{i+1}-$C\alpha^{i+1}$. Values +4°±2° and -179°±2° represent Z and E isomers correspondingly. Populations are calculated over all residues in six families of NMR structures and grouped with 2^0 step.

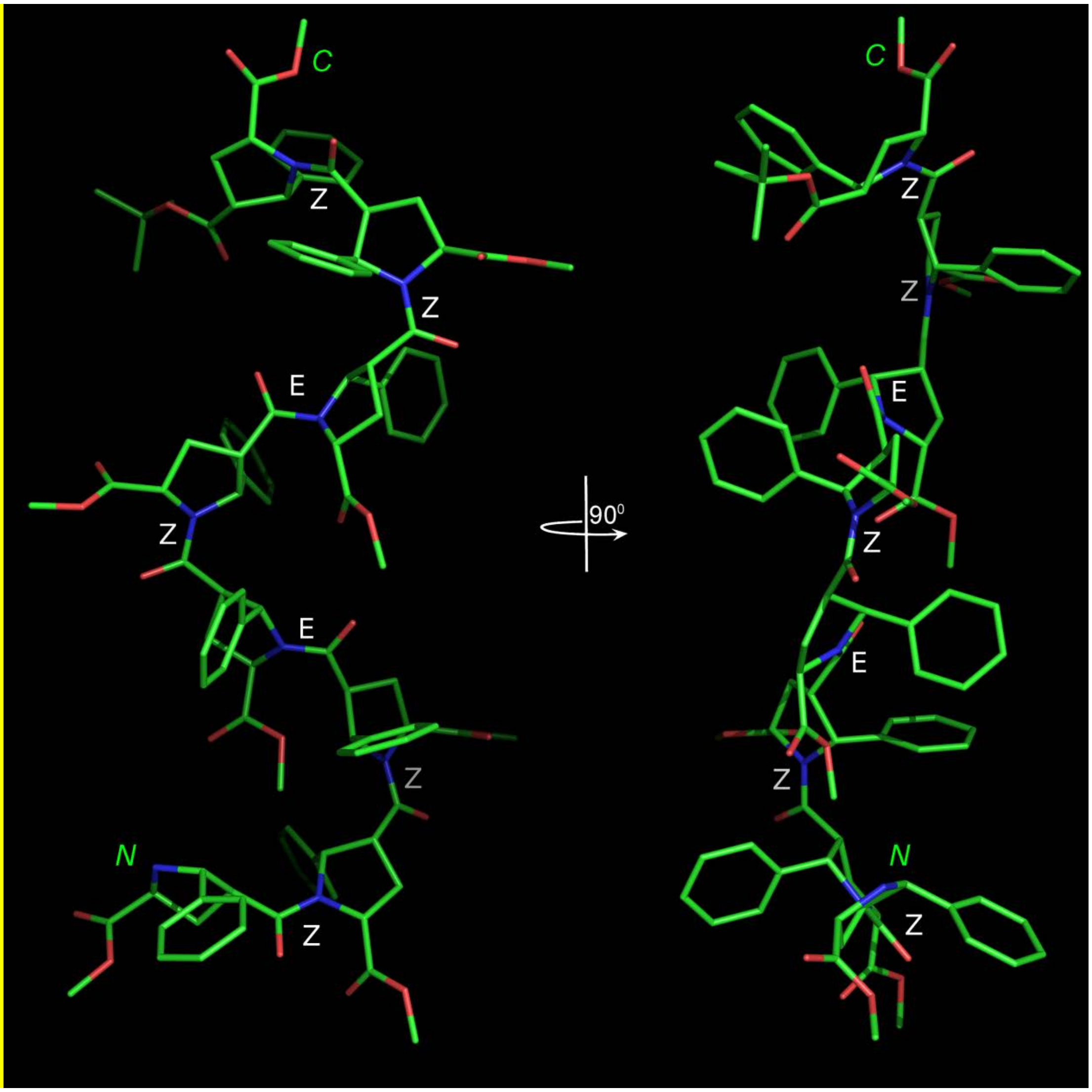

Supplementary Figure 9. The model of the alternating β-proline octamer built using the NMR structures of ZZE tetramer and ZEZZ pentamer.

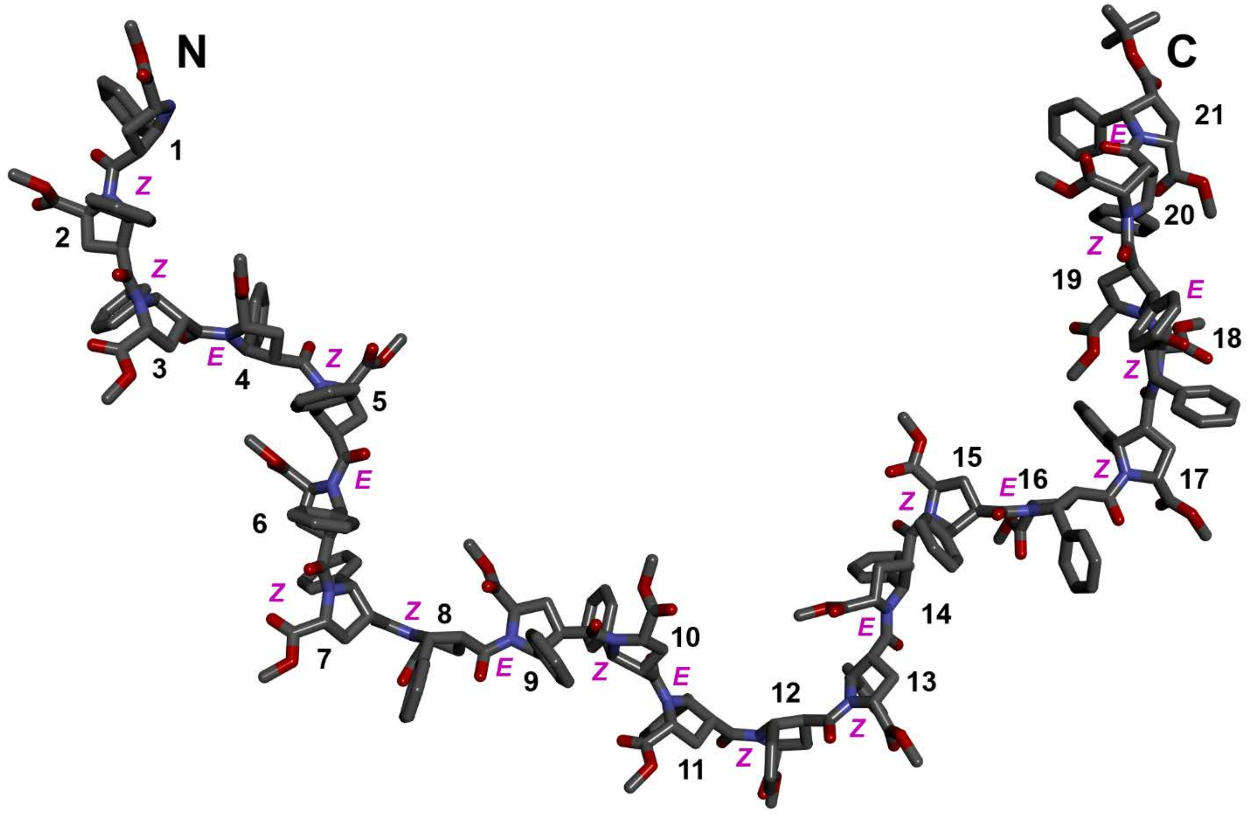

Supplementary Figure 10. Hypothetical model of the alternating β-proline 21-mer with the repetitive (ZZ) and (EZE) fragments.

2 Supplementary Tables

Table S1. Calculated and experimental chemical shifts for (*ZZZ*)-conformation of tetramer the alternating β-proline tetramer **1**.

	atom name	Shielding constant	Shielding constant averaged	Calculated chemical shift, ppm	Exp. shift, ppm	delta	atom name	Shielding constant	Shielding constant averaged	Calculated chemical shift, ppm	Exp. shift, ppm	delta
**Res 1**	Cα	118.22	118.22	64.82	64.16	0.66	N	197.83	197.83			
	Cβ	132.07	132.07	**51.63**	**46.43**	5.20	Hα	26.32	26.32	**5.11**	**4.51**	0.59
	Cγ	152.32	152.32	32.33	33.24	0.91	Hβ	28.29	28.29	3.24	3.02	0.23
	Cδ	124.01	124.01	59.30	58.78	0.53	Hγ1	29.89	29.89	1.74	1.79	0.05
	C	8.45	8.45	169.40	171.24	1.84	Hγ2	29.20	29.20	**2.39**	**1.96**	0.42
	C1	35.32	35.32	**143.80**	**139.75**	4.05	Hδ	27.57	27.57	**3.92**	**3.02**	0.91
	C2*	54.29<br>53.17	53.73	126.26	126.66	0.40	H2*	23.89<br>23.37	23.63	7.65	7.37	0.28
	C3*	52.47<br>52.15	52.31	127.62			H3*	23.97<br>23.91	23.94	7.36	7.33	0.03
	C4	52.61	52.61	127.33			H4	24.00	24.00	7.29		
**Res 2**	Cα	120.81	120.81	62.35	60.92	1.43	N	92.75	92.75			
	Cβ	134.44	134.44	**49.36**	**46.51**	2.86	Hα	25.44	25.44	**5.93**	**5.01**	0.93
	Cγ	155.08	155.08	29.70	28.78	0.92	Hβ	28.16	28.16	**3.37**	**2.68**	0.69
	Cδ	121.52	121.52	**61.67**	**58.18**	3.50	Hγ1	30.06	30.06	1.57	1.55	0.02
	C	12.32	12.32	165.71	166.79	1.08	Hγ2	29.33	29.33	2.26	2.05	0.21
	C1	39.91	39.91	139.43	138.77	0.67	Hδ	28.02	28.02	3.50	3.32	0.19
	C2*	54.22<br>52.75	53.49	126.49	126.30	0.19	H2*	24.12<br>23.28	23.70	7.58	7.43	0.15

		52.27	52.41	127.52			H3*	23.97	23.92	7.37	7.27	0.10
	C3*	52.55						23.87				
	C4	52.99	52.99	126.97			H4	23.96	23.96	7.34		
	Cα	121.22	121.22	61.96	61.33	0.63	N	88.31	88.31			
	Cβ	133.66	133.66	**50.11**	**46.77**	3.35	Hα	25.54	25.54	5.85	5.53	0.32
	Cγ	154.44	154.44	30.31	29.28	1.03	Hβ	28.23	28.23	3.30	3.21	0.10
	Cδ	123.27	123.27	60.01	59.03	0.98	Hγ1	29.00	29.00	**2.58**	**2.00**	0.58
	C	12.10	12.10	165.92	167.32	1.40	Hγ2	29.81	29.81	1.81	1.74	0.06
	C1	40.65	40.65	138.73	138.20	0.52	Hδ	27.89	27.89	3.62	3.36	0.26
		53.94	52.82	127.13	126.64	0.48	H2*	22.61	23.10	8.15	7.60	0.55
	C2*	51.70						23.59				
		51.82	51.64	128.25			H3*	23.77	23.72	7.56	7.47	0.10
	C3*	51.47						23.66				
**Res 3**	C4	52.29	52.29	127.63			H4	23.82	23.82	7.47		
	Cα	119.31	119.31	63.78	62.04	1.74	N	91.59	91.59			
	Cβ	129.76	129.76	**53.83**	**49.38**	4.45	Hα	25.23	25.23	**6.14**	**5.46**	0.67
	Cγ	155.29	155.29	29.50	28.11	1.39	Hβ	27.94	27.94	3.58	3.51	0.06
	Cδ	122.90	122.90	60.36	58.50	1.85	Hγ1	29.46	29.46	2.14	2.29	0.15
	C	9.78	9.78	168.13	167.19	0.95	Hγ2	29.40	29.40	2.19	2.15	0.05
**Res 4**	C1	42.01	42.01	137.43	138.07	0.64	Hδ	27.71	27.71	3.80	3.80	0.01
		52.85	51.81	128.09	127.47	0.62	H2*	23.23	22.86	8.37	7.78	0.60
	C2*	50.77						22.49				
		52.32	52.67	127.27			H3*	23.81	23.78	7.51	7.52	0.01
	C3*	53.03						23.75				
	C4	52.41	52.41	127.51			H4	23.70	23.70	7.58	7.44	0.14

								30.82				
		156.28						30.82				
		161.69	159.88	25.12	27.10	1.98		27.99				
	CT*	161.69						29.59				
							HT*	30.83	30.36	1.29	1.16	0.12
								31.64				
								30.82				
								29.70				

Table S2. Calculated and experimental chemical shifts for (*ZZE*)-conformation of tetramer the alternating β- proline tetramer **1**.

	**atom name**	**Shielding constant**	**Shielding constant averaged**	**Calculated chemical shift, ppm**	**Exp. shift, ppm**	**delta**	**atom name**	**Shielding constant**	**Shielding constant averaged**	**Calculated chemical shift, ppm**	**Exp. shift, ppm**	**delta**
**Res 1**	C	6.34	6.34	171.41	170.13	1.28	HN	29.73	29.73	**1.88**	**3.29**	1.41
	Cα	119.31	119.31	63.78	62.76	1.02	Hα	26.77	26.77	4.68	4.82	0.13
	Cβ	136.20	136.20	47.69	47.00	0.69	Hβ	28.54	28.54	3.01	2.96	0.04
	Cγ	151.73	151.73	32.89	32.54	0.35	Hγ1	29.40	29.40	2.19	2.10	0.09
	Cδ	123.77	123.77	59.53	58.79	0.75	Hγ2	30.00	30.00	1.63	1.74	0.11
	C2*	50.51	51.55	128.34	126.31	2.03	Hδ	27.86	27.86	3.65	3.65	0.00
		52.59					H2*	23.63	23.33	7.93	7.34	0.59
								23.03				
							H3*	24.03	23.76	7.53	7.40	0.13
								23.48				

**Res 2**	C	11.47	11.47	166.52	165.99	0.53	Hα	26.66	26.66	**4.78**	**5.30**	0.52
	Cα	120.85	120.85	62.32	60.47	1.85	Hβ	28.93	28.93	2.64	2.69	0.06
	Cβ	134.78	134.78	49.04	47.07	1.97	Hγ1	30.33	30.33	1.32	1.68	0.36
	Cγ	154.50	154.50	30.25	28.16	2.09	Hγ2	29.48	29.48	2.12	2.02	0.10
	Cδ	121.77	121.77	61.43	57.84	3.59	Hδ	28.54	28.54	3.01	3.04	0.04
	C2*	54.10	53.35	126.63	125.84	0.79	H2*	23.37	23.77	7.51	7.35	0.16
		52.59						24.18				
							H3*	23.88	23.82	7.47	7.42	0.05
								23.75				
**Res 3**	C	10.15	10.15	167.78	167.65	0.13	Hα	25.69	25.69	5.70	5.73	0.02
	Cα	118.64	118.64	64.42	61.84	2.58	Hβ	27.97	27.97	3.55	3.67	0.12
	Cβ	133.79	133.79	49.99	46.78	3.21	Hγ1	29.50	29.50	2.10	2.06	0.05
	Cγ	153.39	153.39	31.31	29.59	1.73	Hγ2	29.40	29.40	2.20	2.23	0.03
	Cδ	123.40	123.40	59.89	58.54	1.35	Hδ	28.00	28.00	3.52	3.76	0.24
	C2*	51.64	52.16	127.76	126.83	0.93	H2*	22.66	23.16	8.09	7.35	0.74
		52.67						23.67				
							H3*	24.02	24.21	7.10	7.04	0.06
								24.40				
**Res 4**	C	5.66	5.66	172.05	168.37	3.68	Hα	25.98	25.98	5.42	5.37	0.05
	Cα	115.45	115.45	67.46	62.88	4.58	Hβ	28.10	28.10	3.43	3.51	0.09
	Cβ	131.39	131.39	**52.27**	**46.56**	5.71	Hγ1	28.70	28.70	2.85	2.72	0.14
	Cγ	150.39	150.39	34.17	31.28	2.89	Hγ2	28.82	28.82	2.75	2.66	0.09
	Cδ	119.05	119.05	**64.03**	**59.12**	4.91	Hδ	26.33	26.33	5.09	5.41	0.32
	C2*	51.90	52.45	127.48	128.19	0.71	H2*	24.18	23.33	7.93	7.34	0.60
		53.00						22.48				

						H3*	24.04	23.75	7.53	7.26	0.27
							23.47				
	160.85						30.43				
CT*	155.76	159.56	25.44	26.92	1.49		30.36				
	162.06						30.68				
							30.11				
						HT*	31.22	30.55	1.11	1.05	0.06
							31.02				
							29.63				
							30.75				
							30.71				

Table S3. Calculated and experimental chemical shifts for (*ZEZ*)-conformation of tetramer the alternating β- proline tetramer **1**.

	**atom name**	**Shielding constant**	**Shielding constant averaged**	**Calculated chemical shift, ppm**	**Exp. shift, ppm**	**delta**	**atom name**	**Shielding constant**	**Shielding constant averaged**	**Calculated chemical shift, ppm**	**Exp. shift, ppm**	**delta**
**Res 1**	C	7.33	7.33	170.47	170.14	0.33	HN	29.94	29.94	**1.68**	**3.21**	1.53
	Cα	116.77	116.77	66.20	62.92	3.28	Hα	27.13	27.13	4.34	4.47	0.13
	Cβ	131.05	131.05	**52.59**	**46.92**	5.67	Hβ	28.59	28.59	2.96	2.87	0.09
	Cγ	147.12	147.12	**37.29**	**32.22**	5.06	Hγ1	29.38	29.38	2.21	2.02	0.19
	Cδ	122.39	122.39	60.84	58.55	2.29	Hγ2	29.89	29.89	1.73	1.69	0.04
	C2*	50.42<br>52.75	51.59	128.30	126.49	1.81	Hδ	28.10	28.10	3.43	3.66	0.23
							H2*	23.93<br>23.93	23.93	7.36	7.18	0.18
							H3*	23.81<br>23.82	23.81	7.48	7.26	0.22
**Res 2**	C	9.58	9.58	168.33	167.46	0.87	Hα	26.67	26.67	**4.78**	**5.24**	0.46
	Cα	119.87	119.87	63.24	61.95	1.30	Hβ	28.73	28.73	2.83	3.14	0.31
	Cβ	137.03	137.03	46.89	46.50	0.39	Hγ1	29.54	29.54	2.06	1.97	0.09
	Cγ	153.36	153.36	31.34	29.88	1.46	Hγ2	29.99	29.99	1.64	1.92	0.28
	Cδ	121.94	121.94	61.27	57.75	3.52	Hδ	28.27	28.27	3.26	3.33	0.07
	C1	40.84	40.84	138.54	138.55	0.01	H2*	23.95<br>23.59	23.77	7.52	7.65	0.13
	C2*	53.17<br>52.54	52.85	127.10	127.15	0.05	H3*	24.39<br>24.08	24.23	7.08	6.97	0.10
	C3*	53.52<br>51.95	52.74	127.21	127.18	0.03						
**Res 3**	C	5.84	5.84	171.89	170.17	1.72	Hα	26.71	26.71	**4.73**	**5.27**	0.53

	Cα	115.33	115.33	67.57	64.64	2.94	Hβ	28.57	28.57	2.98	3.11	0.13
	Cβ	137.29	137.29	46.65	44.56	2.09	Hγ1	29.30	29.30	2.29	2.20	0.09
	Cγ	148.58	148.58	**35.90**	**31.72**	4.18	Hγ2	29.52	29.52	2.08	2.38	0.30
	Cδ	118.97	118.97	64.11	60.14	3.97	Hδ	26.88	26.88	**4.57**	**5.18**	0.60
	C1	38.84	38.84	140.45	138.56	1.89	H2*	23.83<br>23.46	23.65	7.63	7.40	0.24
	C2*	53.45<br>51.40	52.43	127.50	127.31	0.19	H3*	23.96<br>23.81	23.89	7.41	7.75	0.34
**Res 4**	C	10.00	10.00	167.92	166.88	1.04	Hα	26.79	26.79	4.66	4.73	0.07
	Cα	120.01	120.01	63.11	61.48	1.63	Hβ	29.23	29.23	2.36	2.34	0.02
	Cβ	130.78	130.78	**52.85**	**48.80**	4.05	Hγ1	29.86	29.86	1.76	2.01	0.25
	Cγ	155.69	155.69	29.12	27.48	1.64	Hγ2	29.76	29.76	1.85	1.93	0.08
	Cδ	122.26	122.26	60.97	58.08	2.89	Hδ	28.09	28.09	3.43	3.45	0.02
	C1	42.08	42.08	137.36	137.73	0.36	H2*	23.56<br>23.67	23.62	7.66	7.57	0.09
	C2*	48.20<br>52.45	50.32	129.51	127.81	1.70	H3*	24.32<br>23.56	23.94	7.35	7.34	0.02
	CT*	161.82<br>156.55<br>161.54	159.97	25.04	27.00	1.96	HT*	29.88<br>31.76<br>31.04<br>30.90<br>29.72<br>30.85<br>30.75<br>30.94	30.75	0.92	1.10	0.18

30.88

Table S4. Calculated and experimental chemical shifts for (*EZZ*)-conformation of tetramer the alternating β- proline tetramer **1**.

	**atom name**	**Shielding constant**	**Shielding constant averaged**	**Calculated chemical shift, ppm**	**Exp. shift, ppm**	**delta**	**atom name**	**Shielding constant**	**Shielding constant averaged**	**Calculated chemical shift, ppm**	**Exp. shift, ppm**	**delta**
**Res 1**	C	6.02	6.02	171.71	172.04	0.33	Hα	26.96	26.96	4.50	4.20	0.30
	Cα	121.07	121.07	62.10	63.99	1.88	Hβ	28.11	28.11	3.41	3.29	0.12
	Cβ	133.20	133.20	**50.55**	**45.34**	5.21	Hγ1	28.98	28.98	2.59	2.41	0.18
	Cγ	153.66	153.66	31.06	34.38	3.32	Hγ2	29.80	29.80	1.82	1.92	0.10
	Cδ	124.50	124.50	58.83	58.40	0.43	Hδ	27.66	27.66	3.84	3.85	0.01
							H2*	23.86<br>24.43	24.15	7.16	7.41	0.25
							H3*	24.69<br>24.13	24.41	6.91	7.11	0.20
**Res 2**	C	12.01	12.01	166.01	168.89	2.87	Hα	25.65	25.65	5.74	5.50	0.23
	Cα	121.11	121.11	62.07	62.79	0.72	Hβ	28.71	28.71	2.85	3.12	0.27
	Cβ	136.80	136.80	47.12	44.19	2.93	Hγ1	29.79	29.79	1.83	2.06	0.23
	Cγ	152.71	152.71	31.96	31.84	0.13	Hγ2	28.79	28.79	**2.77**	**2.30**	0.47
	Cδ	120.04	120.04	63.09	59.04	4.04	Hδ	27.13	27.13	**4.34**	**4.76**	0.42
	C2*	53.22<br>52.87	53.04	126.92	126.17	0.75	H2*	24.44<br>22.80	23.62	**7.66**	**7.20**	0.46
							H3*	24.44<br>23.81	24.12	7.18	6.95	0.23
**Res 3**	Cα	121.15	121.15	62.03	60.08	1.95	Hα	25.71	25.71	5.68	5.70	0.02
	Cβ	135.44	135.44	48.41	46.69	1.72	Hβ	28.35	28.35	3.18	2.87	0.31

	Cγ	154.37	154.37	30.38	28.84	1.54	Hγ1	29.02	29.02	**2.55**	**1.98**	0.58
	Cδ	124.73	124.73	58.62	58.17	0.45	Hγ2	29.65	29.65	**1.96**	**1.51**	0.45
							Hδ	28.26	28.26	3.27	2.99	0.27
								22.76				
							H2*		23.35	7.92	7.60	0.32
								23.93				
								23.84				
							H3*		23.86	7.43	7.35	0.09
								23.88				
	Cα	119.76	119.76	63.35	61.10	2.25	Hα	25.56	25.56	5.82	5.90	0.08
	Cβ	131.16	131.16	52.49	49.63	2.86	Hβ	27.93	27.93	3.58	3.58	0.00
	Cγ	155.14	155.14	29.64	28.39	1.26	Hγ1	29.55	29.55	2.05	2.32	0.27
**Res 4**	Cδ	123.11	123.11	60.16	58.78	1.38	Hγ2	29.54	29.54	2.06	2.15	0.09
							Hδ	27.73	27.73	3.78	3.78	0.00
								23.61				
							H2*		23.10	**8.15**	**7.73**	0.41
								22.60				
								23.92				
							H3*		23.83	7.46	7.44	0.02
								23.74				
		156.22						30.75				
	CT*	160.39	159.33	25.65	27.05	1.39		30.60				
		161.37						29.93				
								30.23				
							HT*	30.00	30.30	1.34	1.12	0.23
								30.56				
								30.55				
								30.62				
								29.46				
